# Green edible crataegi fructus-derived carbon dots ameliorate hyperlipidemia via the PPARα signaling pathway

**DOI:** 10.3389/fphar.2026.1985522

**Published:** 2026-10-16

**Authors:** Guojiao Shang, Yafang Zhao, Ertong Dai, Ruiyan Liu, Minlong Xia, Zixuan Lu, Siqi Wang, Bilin Jin, Herui Huang, Yue Zhang, Hui Kong, Huihua Qu, Yan Zhao

**Affiliations:** 1 School of Traditional Chinese Medicine, Beijing University of Chinese Medicine, Beijing, China; 2 School of Traditional Chinese Medicine, Tianjin University of Traditional Chinese Medicine, Tianjin, China; 3 School of Life Sciences, Beijing University of Chinese Medicine, Beijing, China; 4 Center of Scientific Experiment, Beijing University of Chinese Medicine, Beijing, China

**Keywords:** carbon dots, crataegi fructus, hyperlipidemia, nanomedicine, PPARα signaling pathway

## Abstract

**Background:**

Hyperlipidemia (HL) is a lipid metabolism disorder that contributes significantly to the global burden of metabolic diseases. Hence, novel pharmacological interventions are urgently needed. Our study explored the potential mechanisms by which Crataegi Fructus Carbonisata carbon dots (CFC-CDs) act against hyperlipidemia through *in vivo* and *in vitro* experiments.

**Methods:**

We prepared CFC-CDs and comprehensively characterized them. Subsequently, we assessed the effects of CFC-CDs on serum lipid profiles in a high-fat diet-induced rat model and used transcriptomic analysis to explore the potential mechanisms underlying their lipid-lowering effects. Additionally, we developed an *in vitro* model of sodium oleate (OA)-induced lipid deposition in HepG2 cells and validated it using the PPARα inhibitor GW6471.

**Results:**

The CFC-CDs had an ultrasmall size ranging from 1.34 to 4.67 nm and an interplanar spacing of approximately 0.210 nm. Their surfaces contained functional groups, including–OH, C=O, and C=N. These CDs exhibited excellent biocompatibility. Animal studies demonstrated that oral treatment with CFC-CDs markedly reduced high-fat diet (HFD)-induced hyperlipidemia. Liver transcriptome analysis indicated that CFC-CDs exerted lipid-lowering effects primarily by activating the PPARα signaling pathway, suggesting that CFC-CDs have significant metabolic reprogramming capabilities. CFC-CDs also exhibited excellent lipid-lowering effects *in vitro*, and the PPARα inhibitor GW6471 reversed the protective effects of CFC-CDs, thereby leading to lipid accumulation. CFC-CDs may be a potential therapeutic agent for HL.

**Conclusion:**

The above evidence suggests that CFC-CDs synthesized by one-step calcination can significantly improve lipid metabolism disorders. Our study shows that CFC-CDs are a biomass-derived carbonaceous nanomaterial exhibiting effective lipid-lowering properties and broadening their potential clinical applications. This research provides crucial insights into the use of CFC-CDs in metabolic disease treatment and lays a solid experimental foundation for developing a new lipid-lowering candidate.

## 1 Introduction

A prevalent metabolic disorder, hyperlipidemia (HL) features abnormal circulating lipid levels: elevated TC, TG, and LDL-C, together with diminished HDL-C (Berisha et al., 2025; Bian et al., 2024; Cheng et al., 2024). The incidence of hyperlipidemia is rising globally (Liu et al., 2022). The number of cases of this disease is growing in China due to changing lifestyles.

Hyperlipidemia is a major risk factor for cardiovascular disease (Akbarpour et al., 2025), and hyperlipidemia-induced atherosclerotic cardiovascular disease (ASCVD) has become a leading cause of death globally (Zhang Z. et al., 2025; Sandesara et al., 2019). At present, statins, fibrates, PCSK9 inhibitors, and other drugs are the main treatments (Davignon, 2004), supplemented by dietary intervention and lifestyle modification. The management of hyperlipidemia still faces many challenges, and the burden of the disease is heavy, resulting in 4.4 million deaths worldwide each year, and the medical costs related to the management of ASCVD are huge (Tian et al., 2024). It is crucial to explore innovative, safe, and effective treatments for hyperlipidemia to ease the disease’s effects.

The treatment of hyperlipidemia is undergoing a paradigm shift from traditional passive lipid removal to a modern active regulation approach. The mechanism central to PPARα-mediated reprogramming of lipid metabolism involves boosting the transcriptional levels of CPT1A, ACOX1, and other genes associated with β-oxidation (Zhang J. et al., 2023), thereby facilitating fatty acid transport into mitochondria for β-oxidation and, in turn, reducing lipid accumulation in both the liver and the circulation. By regulating the metabolism of key organs such as the liver, PPARα fundamentally restores lipid homeostasis.

Recently, the emergence of carbon dots (CDs) has furnished new concepts for hyperlipidemia treatment (Lin et al., 2025). CDs, a new category of carbon nanomaterials, typically refer to fluorescent nanoparticles under 10 nm in size (Kang et al., 2021; Zeng et al., 2024). CDs exhibit distinctive optical characteristics and find broad application in biomedicine, including bioimaging and disease diagnosis (Xia et al., 2019; Velu and Yoon, 2022; Zhang et al., 2024; González and Merkoçi, 2025; Zhang Y. et al., 2025; Park et al., 2025). A core-shell configuration is exhibited by CDs, wherein the carbon core is enriched with sp^2^-hybridized zones and the surface bears many functional groups - e.g., OH, COOH, C=O, and NH2 (Liu Y. et al., 2024). The diverse surface features of CDs confer a broad spectrum of bioactive capabilities. CDs possess the unique ability to retain their inherent biological activity and chemical structure. This results in the drugs gaining new or enhanced therapeutic properties, reflecting the traditional Chinese medicine concept of combining “preservation” with “transformation” in drug preparation.

CDs generated from plants have garnered interest for improving the delivery and bioavailability of bioactive compounds (Meng et al., 2025; Liu et al., 2025). As a typical representative of the concept of “food and drug homology” (Zhou et al., 2025), Crataegi Fructus has exhibited notable biological actions, including glucose-lowering effects (Lu et al., 2019), anti-inflammatory, and digestive-promoting effects due to its abundance of polyphenols, such as chlorogenic acid, caffeic acid, and quercetin (Zhang R. et al., 2025; Sun et al., 2024). Crataegi Fructus is abounds in organic acids and polysaccharides, which supply ample reactive sites for carbon dot synthesis. As Crataegi Fructus undergoes further carbonization, the levels of its key components, such as organic acids and flavonoids decline dramatically, leading to varied properties and effects. Clinically, roasted Crataegi Fructus is commonly employed to lower blood lipid levels due to the necessity for patients with hyperlipidemia to take medication over an extended period. Raw Crataegi Fructus has a higher concentration of organic acids, which can be irritating to the gastrointestinal tract. Roasted Crataegi Fructus contains fewer organic acids and is more effective in reducing blood lipids. After being roasted, the active ingredients in Crataegi Fructus are easier to extract during brewing. The altered composition of the active ingredients after processing may be directly related to the temperature and time of the carbonation. Our team has synthesized various CDs sourced from Chinese herbal medicine. These CDs have been found to exhibit a range of biological functions in addition to their pharmacodynamic effects prior to nanonization (Lu et al., 2021; Luo et al., 2021; Zhang M. et al., 2023), such as acute ischemic stroke (Zhang Y. et al., 2021), acute myocardial infarction (Dong L. et al., 2024), psoriasis (Zhang M. et al., 2021), and anxiety (Chen et al., 2022). These studies support the clinical use of traditional Chinese medicine (TCM) while also offering strategic and conceptual inputs to move modern TCM development forward.

Here, we examined the lipid-lowering and hepatoprotective effects of CFC-CDs through *in vitro* and *in vivo* experiments. We established a model of lipid accumulation and hepatic steatosis induced by a high-fat diet (HFD). CFC-CDs were administered continuously for 7 weeks. Through histopathological analysis, RNA sequencing, and assessment of lipid metabolism and PPARα-related protein expression, we found that CFC-CDs alleviated lipid metabolic disorders in the liver. We further found that CFC-CDs improved liver function in rats via the PPARα signaling pathway. *In vitro* studies showed that the PPARα inhibitor GW6471 reversed the protective effects of CFC-CDs, leading to excessive lipid accumulation. Therefore, CFC-CDs may be a therapeutic agent for HL.

## 2 Materials and methods

### 2.1 Drugs and chemical reagents

Crataegi Fructus herbal pieces (batch number:C367240302) were sourced from Anxing Traditional Chinese Medicine Slices Co., Ltd. in Anguo City, Hebei Province. The muffle furnace (TL0612) was obtained from Beijing Zhongke Aobo Technology Co., Ltd. The 1 kDa dialysis membrane was obtained from Beijing Ruida Henghui Technology Development Co., Ltd. Phosphatase inhibitors, protease inhibitors, the BCA protein concentration assay kit, and the CCK-8 assay kit were acquired from Beijing LABLEAD Trading Co., Ltd. We bought Oil Red O staining solution from Beijing Solarbio Technology Co., Ltd., and Nanjing Jiancheng Biological Engineering Research Institute supplied the kits for detecting TC and TG. Sodium oleate, a high-fat cell culture supplement, was purchased from Xi’an Kunchuang Technology Co., Ltd., and consumables required for PCR experiments were purchased from Accurate Biotechnology Co., Ltd. The Omni-Easy One-Step PAGE Gel Rapid Preparation Kit, ECL Ultra-Sensitive Chemiluminescent Detection Solution and the fluorescent secondary antibodies (GF555 conjugated to goat anti-rabbit IgG, and BF488 conjugated to goat anti-mouse IgG) were all purchased from Shanghai Yamay Biomedical Technology Co., Ltd. The primary antibodies PPARα, PGC-1α, CPT1A, FABP1, and ACOX1 were purchased from Wuhan Sanying Biotechnology Co., Ltd. The PPARα inhibitor GW6471 (HY-15372) was purchased from the MedChemExpress website. Deionized water was used throughout the experiment.

### 2.2 Preparation of CFC-CDs

The experimenter weighed out 200 g of Crataegi Fructus pieces, placed them in a crucible, covered the crucible with aluminum foil, and then placed the crucible in the muffle furnace. After closing the furnace door, the automatic heating program for the muffle furnace was set as follows: Stage 1: The temperature was ramped to 70 °C over 5 min and held for 25 min; Stage 2: Set the temperature to B°C for 25 min, then held for I hour. Once the calcination program was complete, the experimenter waited until the display indicated that the furnace temperature had dropped to 70 °C. The furnace door was then opened, and the sample was allowed to cool to room temperature. The charred powder was weighed, and the carbonization rate was calculated to be 37.5%. The charred Crataegi Fructus was ground into a powder, mixed with deionized water equal to 30 times the weight of the powder, and decocted in a water bath at 100 °C for 1 h. This decoction process was repeated three times. After each decoction, the solution was allowed to cool to room temperature, filtered coarsely through filter paper, and then fine-filtered through a 0.22 μm microporous membrane. The filtrates from the three decoctions were collected and concentrated using a rotary evaporator. Finally, the combined filtrate was concentrated to 1 g/mL—equivalent to adding 1 g of charred Crataegi Fructus powder per milliliter of the final solution—and the concentrated solution was transferred to a dialysis bag with a MWCO of 1,000 Da. The dialysis bag was placed in a beaker containing deionized water (DW), and the beaker was placed on a magnetic stirrer for dialysis. The DW in the beaker was replaced regularly each day, and dialysis was continued for 7 days, until the DW outside the dialysis bag became colorless and transparent. The liquid inside the dialysis bag was filtered again through a 0.22 μm microporous membrane, and the filtrate was collected and stored at 4 °C for later use.

### 2.3 Characterization of CFC-CDs

We used a Tecnai G2 20 from FEI and a JEM-1230 from Japan Electronic Optics Laboratory to study the morphology and size distribution of CFC-CDs by high-resolution TEM. To compare the chemical composition of Crataegi Fructus and CFC-CDs, we used an Agilent 1,260 Infinity HPLC system. The elemental composition of CFC-CDs was analyzed by a Thermo Fisher Scientific 250 XI XPS spectrometer. The crystal structure of CFC-CDs was examined by X-ray diffraction (D8 Advance DaVinci, Bruker). The optical properties of CFC-CDs were evaluated via fluorescence spectrophotometry (Thermo Fisher Scientific F-4500) and UV-Vis spectrophotometry (CECIL, UK). An FTIR instrument (Nicolet iS, Thermo Fisher Scientific) was employed to assess the functional groups of CFC-CDs, with the spectrum recorded from 400 to 4,000 cm^-1^. Finally, we measured the zeta potential of CFC-CDs using a Zetasizer Nano ZS90 (Malvern).

### 2.4 Grouping, modeling, and administration of experimental animals

Male specific pathogen-free (SPF) Sprague-Dawley (SD) rats, 5 weeks old, were purchased from Beijing Vital River Laboratory Animal Technology Co., Ltd. The ethics review number for this study was BUCM-2024071702-3052. The rats were kept at the Animal Experimentation Center of Beijing University of Chinese Medicine. All rats were housed under quiet conditions at 24.0 °C ± 1.0 °C and 55%–60% relative humidity. The lighting followed a 12-h light/dark cycle. During the study, rats had free access to food and water. The high-fat diet was obtained from Beijing Sibeifu Biotechnology Co., Ltd., with production license number SCXK (Beijing) 2024-0001. This specialized diet consisted of 58.6% basal diet, 15% lard, 20% sucrose, 5% casein, 1.2% cholesterol, and 0.2% sodium cholate, and was kept refrigerated at 4 °C.

According to the 2020 edition of the Pharmacopoeia of the People’s Republic of China, the recommended clinical dosage of Crataegi Fructus is 9–12 g/day. Chinese Materia Medica recommends a decoction dosage of 10–15 g, with a maximum of 30 g. The actual dosage should be adjusted based on the patient’s condition. In this study, a dose of 20 g/day was selected (based on a human body weight of 60 kg); after conversion to an equivalent dose for rats based on body surface area, the rat dosage was 2 g/kg/day. An equal dose of raw Crataegi Fructus was carbonized to produce Crataegi Fructus charcoal, with a carbonization yield of 37.5%. Based on the mass of the Crataegi Fructus charcoal, a CFC-CDs solution at 1 g/mL was prepared and then freeze-dried to obtain CFC-CDs powder. Based on the calculations, after equivalent conversion from the crude drug to Crataegi Fructus charcoal, the corresponding high dose of CFC-CDs powder administered to rats was 1.88 mg/kg/day. After serial dilutions, the medium and low doses were determined to be 0.94 mg/kg/day and 0.47 mg/kg/day, respectively.

Forty-eight SD rats were randomly divided into six groups of eight rats each according to body weight after a 1-week acclimatization period. The rats were divided into six groups: control, HFD, ATV (2 mg/kg), CFC-CDs-H, CFC-CDs-M, and CFC-CDs-L. The control group was given a normal diet, and the other groups were given a high-fat diet for seven consecutive weeks. The control and HFD groups simultaneously received normal saline, while the treatment groups received their respective drugs once daily. After the final gavage, all rats were fasted for 12 h. Sodium pentobarbital was administered by intraperitoneal injection before sample collection.

### 2.5 General situation observation

Weekly body weight recordings and observations of mental outlook, hair condition, food intake, and water intake were conducted during the experiment. We measured body length, specifically from the tip of the nose to the anus, and calculated Lee’s index, using Formula 1:

$$\text{Lees index}=\left[\text{body weight }\left(g\right)\left(1/3\right)*1,000/\text{body length }\left(\text{cm}\right)\right].$$ (1)

At sampling, the liver was weighed, and the organ index was calculated using Formula 2:

Organ index=organ weight/body weight×100%. (2)

Organ weight, g; Body weight, g.

### 2.6 Measurement of blood lipids and liver function

We collected blood samples from the abdominal aorta of anesthetized rats; the samples were kept at room temperature for 4 hours to allow coagulation. Subsequently, to separate serum, the samples were centrifuged at 3500 rpm for 15 min at 4 °C. We then used an automatic serum biochemical analyzer to measure a series of biochemical markers in the collected serum, including TC, TG, LDLC, HDLC, ALT, AST, and TBA.

### 2.7 Histopathological analysis

For H&E staining, rat liver tissue was fixed in 4% paraformaldehyde, dehydrated, embedded in paraffin, and cut into 5 μm sections for histological observation. Meanwhile, 9-μm-thick frozen liver sections were stained with Oil Red O to detect lipids. Adipose tissue underwent identical preparation. All sections were microscopically analyzed (IX73, Olympus, Japan) and photographed for future analysis. The section evaluation of histopathological staining (H&E, Oil Red O) was scored by two independent pathological researchers who were unaware of the group assignment in a double-blind manner.

### 2.8 Liver lipids and serum testosterone levels were detected by ELISA

We used the supernatant of the liver tissue homogenate as the sample and measured the levels of TC and TG. The serum testosterone concentration of rats was measured using a testosterone quantitative detection kit, and all specific operation steps were strictly performed according to the kit’s instructions.

### 2.9 Biocompatibility of CFC-CDs

For the hemolysis assay, fresh rat red blood cells were employed to assess the hemolytic effects of CFC-CDs. Moreover, the *in vivo* biocompatibility was assessed by serum biochemistry and H&E staining.

This study utilized SPF-grade Kunming mice as experimental subjects. Beijing Vital River Laboratory Animal Technology Co., Ltd. provided the animals, and the ethical approval number for this experiment was BUCM-2023122202-4,251. After an adaptation period, mice were randomly allocated to groups according to body weight: the experimental group received CFC-CDs (8 mg/kg, approximately 4–5 times the daily therapeutic dose) by gavage once daily, while the control group received an equal volume of normal saline for the same duration. The body weight of mice was monitored and recorded every 2 days. After anesthesia, blood was collected from the orbital sinus, and the color, shape, and size of the major organs, as well as any bleeding, were observed. The tissues were subsequently fixed in 4% paraformaldehyde for further pathological examination.

### 2.10 Transcriptome sequencing of liver tissue

In this experiment, we extracted high-quality total RNA from biological samples using the Trizol-chloroform method, assessed RNA quality, and constructed a cDNA library. The Illumina NovaSeq 6000 platform was used for sequencing, and DESeq2 and other tools were used to screen differentially expressed genes (DEGs). Threshold values (e.g., padj <0.05, |log_2_ (fold change)| ≥ 0.585) were set, and the KOBAS database and clusterProfiler software were used to analyze KEGG pathway enrichment of DEGs.

### 2.11 Cell culture

Human hepatocellular carcinoma cells (HepG2) were acquired from Procell Biotechnology Co., Ltd. The cells were cultured at 37 °C in a 5% CO_2_ incubator in medium supplemented with 10% fetal bovine serum (Corning, USA) and 1% penicillin/streptomycin.

### 2.12 Oleic acid-induced lipid accumulation model in HepG2 cells

HepG2 cells were exposed to 400 μM OA to induce an *in vitro* hyperlipidemia model. After 6 h of pretreatment with CFC-CDs, HepG2 cells received OA stimulation for 24 h. Experimental groups included control, OA, H (500 μg/mL), M (250 μg/mL), and L (125 μg/mL). For inhibitor experiments, HepG2 cells were plated in 12-well plates at an appropriate density. Cells received a 10-μM GW6471 pretreatment (HY-15372, MCE) (Xi et al., 2021) for 2 h before OA induction. The experiment comprised four groups: control, OA, OA+ H, and OA+ H + GW6471.

### 2.13 Cell viability was detected by the CCK8 assay

We used the CCK8 assay to evaluate how CFC-CDs affect HepG2 cell viability. Formula 3 was used to calculate cell viability:

$$\text{cell viability }\left(\%\right)=\left(\text{Ae}-\text{Ab}\right)/\left(\text{Ac}-\text{Ab}\right)\times 100,$$ (3)

where Ae, Ab, and Ac = absorbance at 450 nm of experimental, blank, and control groups, respectively.

### 2.14 Oil red O staining

Staining with the Solarbio Oil Red O kit involved fixing cells in 4% paraformaldehyde (20 min), washing with PBS, treating with 60% isopropanol, and staining in the dark for 15 min, washing, counterstaining with hematoxylin, sealing with glycerin gelatin, and observing microscopically.

### 2.15 TC,TG levels

Triglyceride (TG) and total cholesterol (TC) content in cell lysates were determined using commercially available kits following the manufacturer’s instructions.

### 2.16 BODIPY 493/503 fluorescent staining

Cells were fixed with 4% paraformaldehyde according to the BODIPY 493/503 instructions, then incubated with the working solution (Solarbio, CA5090) for 15-30 min at room temperature in the dark. After nuclear staining with Hoechst 33342, green fluorescent lipid droplets were visualized under a fluorescence microscope.

### 2.17 Immunofluorescence

HepG2 cells were cultured in 12-well plates and fixed (4% paraformaldehyde, 15 min) after drug intervention. The cells were permeabilized with 0.2% Triton X-100 for 20 min and blocked (5% BSA, 60 min). HepG2 cells were incubated with anti-PPARα, anti-PGC-1α, anti-CPT1A, anti-ACOX1, and anti-FABP1 (diluted 1:200) at 4 °C overnight, followed by incubation with a fluorescent secondary antibody for 60 min and nuclear staining with DAPI (1 μg/mL) for 5 min. Images were captured using a fluorescence microscope (Olympus IX73).

### 2.18 RT-qPCR

We extracted RNA from liver tissue samples using the SteadyPure universal RNA extraction kit (AG21017) to ensure proper concentration and integrity, and then synthesized cDNA with a reverse transcription kit. The amplification system was 20 μL, and the amplification was performed with a fluorescence quantitative PCR apparatus (Bio-Rad, USA), after which the Ct value was determined. The relative mRNA expression levels between groups were analyzed by the 2^(−ΔΔCt)^ method and normalized to the level of β-actin in the same sample. Primer sequences are listed in Table 1.

**TABLE 1:** Primer sequences of target genes.

Gene name	Primer sequences
FABP1-F	GGA​AAA​GGT​CAA​GGC​AGT​GG
FABP1-R	GTA​GAC​GAT​GTC​ACC​CAG​TGT
CPT1A-F	CAG​GCC​GAA​AAC​CCA​TGT​TG
CPT1A-R	AGG​CCT​CAC​CGA​CTG​TAG​AT
PPARα-F	GGC​AAT​GCA​CTG​AAC​ATC​GAG
PPARα-R	GCC​GAA​TAG​TTC​GCC​GAA​AG
PGC-1α-F	CCG​TAG​GCC​CAG​GTA​TGA​CA
PGC-1α-R	CTC​TCA​GTT​CTG​TCC​GCG​TT
ACOX1-F	CCT​TTG​TTG​TCC​CTA​TCC​GTG​AA
ACOX1-R	CAG​GTA​GCC​GTT​ATC​CAT​CTC​TTC
β-actin-F	GGA​GAT​TAC​TGC​CCT​GGC​TCC​TA
β-actin-R	GAC​TCA​TCG​TAC​TCC​TGC​TTG​CTG

### 2.19 Western blot

We extracted proteins from liver tissue via lysis, homogenization, and centrifugation, and then quantified them using the BCA kit. After denaturation by boiling, proteins were electrophoresed and transferred to PVDF membranes. The membranes were blocked with 5% skim milk for 2 h and then incubated overnight at 4 °C with the corresponding primary antibodies. The primary antibodies used in this study were anti-PPARα (1:1,000, 66826-1-1G, Proteintech), anti-CPT1A (1:1,000, 15184, Proteintech), anti-PGC-1α (1:8000, 1C1B2, Proteintech), anti-ACOX1 (1:6000, 10957, Proteintech), anti-FABP1 (1:8000, 13626, Proteintech), and anti-β-actin (1:8000, Proteintech). After three TBST washes, the membranes were incubated for 1 h at room temperature with HRP-conjugated affinity-purified goat anti-rabbit or anti-mouse IgG (H + L) secondary antibodies (1:10000). ECL luminescent substrate was prepared, and images were acquired using an imaging system (Tanon 5200, China). Using β-actin for normalization, we calculated the relative protein expression levels in each group. ImageJ software was used for data analysis to calculate the relative expression levels of target proteins. The procedure for cell protein extraction was the same as that for the animal experiments.

### 2.20 Statistical analysis

Data were analyzed with SPSS 26.0. All quantitative data were assessed for normality by the Shapiro-Wilk test and homogeneity of variance by Levene’s test. Data with normal distribution and homogeneity of variance were compared among multiple groups using one-way ANOVA followed by Tukey’s HSD *post hoc* test. If the variances were not uniform, Welch’s ANOVA with Games-Howell *post hoc* test was used. For non-normally distributed data, the nonparametric Kruskal–Wallis test combined with Dunn’s multiple comparison test was used. All results were presented as mean ± standard deviation. P < 0.05 indicated that the difference was statistically significant.

## 3 Results

### 3.1 Characterization of CFC-CDs prepared by optimal process

Based on previous experiments, we found that CFC-CDs prepared at B °C for I hour exhibited excellent lipid-lowering effects. In this study, we selected the optimal preparation conditions to synthesize CFC-CDs. Low- and high-resolution TEM revealed the morphology of CFC-CDs. CFC-CDs were spherical nanoparticles with a regular shape, uniform size, and excellent dispersion. The particle size of CFC-CDs varied from 1.34 to 4.67 nm, with an average of (2.56 ± 0.69) nm. HR-TEM revealed that the lattice spacing was 0.210 nm (Figures 1A,B). Next, using DLS, the dispersion and stability of CFC-CDs were measured with a Malvern nanoparticle size analyzer. The polydispersity index (PDI) was used to describe the uniformity of the particle size distribution. The DLS results showed the PDI values of CFC-CDs in water, DMEM, and PBS were 0.213, 0.213, and 0.301, respectively (Figure 1C), indicating that the solutions exhibited good overall stability and dispersion.

**FIGURE 1:**
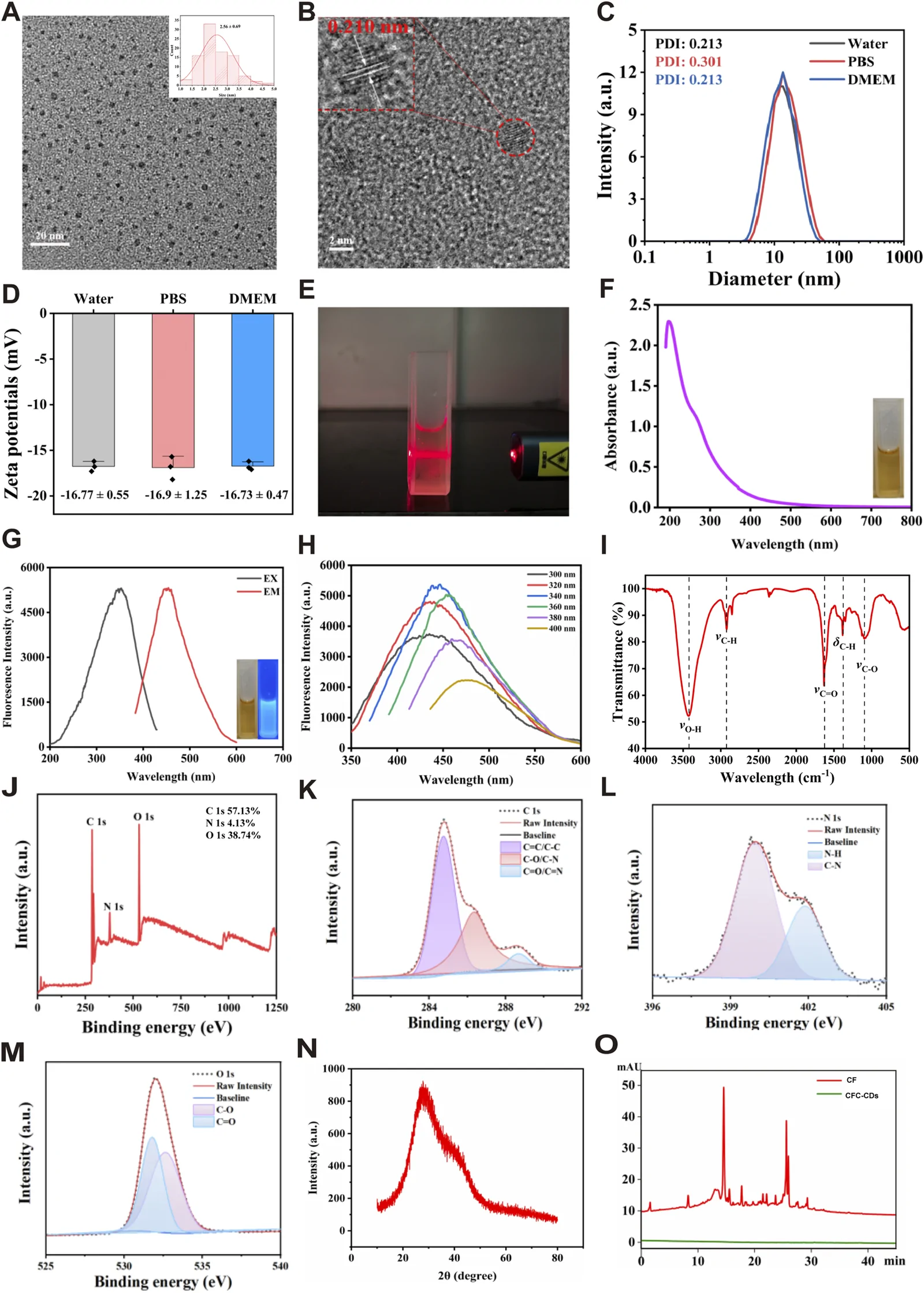
Synthesis and characterization of CFC-CDs. **(A)** TEM image of CFC-CDs and histogram depicting their particle size distribution. Scale bar: 20 nm. **(B)** HR-TEM images of CFC-CDs. Scale bars: 2 nm. **(C)** DLS analysis of CFC-CDs in water, PBS, and DMEM. **(D)** Zeta potential of CFC-CDs in water, PBS, and DMEM (n = 3). **(E)** Tyndall effect of CFC-CDs. **(F)** UV-Vis absorption spectrum of CFC-CDs. **(G)** Fluorescence excitation (black) and emission (red) spectra of CFC-CDs. **(H)** Fluorescence spectra of CFC-CDs at various excitation wavelengths. **(I)** FTIR spectrum of CFC-CDs. **(J–M)** XPS spectra of CFC-CDs: C 1s, N 1s, and O 1s. **(N)** XRD pattern of CFC-CDs. **(O)** HPLC analysis of CFC-CDs.

The zeta potential of CFC-CDs was measured in three physiologically relevant media: water, PBS, and DMEM. In water, the zeta potential was −16.77 ± 0.55 mV; in PBS, it was −16.9 ± 1.25 mV; and in DMEM, it was −16.73 ± 0.47 mV (Figure 1D). These results showed that the zeta potentials of CFC-CDs in the three media were all negative, indicating that CFC-CDs carried a negative charge in these media. This negative charge was related, on the one hand, to the carboxyl and hydroxyl functional groups on CFC-CDs and, on the other hand, to the neutral or weakly basic nature of the media. The presence of a negative charge helps prevent aggregation between particles, thereby improving their stability in solution, and also helps enhance biocompatibility and reduce toxicity. The zeta potential values of CFC-CDs across the three media were similar, indicating that the surface charge properties of CFC-CDs were relatively stable.

The optical properties of CFC-CDs were additionally evaluated. The Tyndall effect frequently manifests in nanoparticle solutions smaller than 100 nm (Hada et al., 2024), because the ultra-small size of nanoparticles scatters visible light; therefore, the Tyndall effect is used to evaluate the stability of nanoparticle colloidal solutions in experiments. When the CFC-CDs dispersion was irradiated vertically with a red laser source (650 nm), a clear, straight, narrow light path could be observed (Figure 1E), indicating the existence of a stable colloidal phase in the system.

CFC-CDs exhibited a distinctive light absorption profile in the UV-visible spectrum, typically in the ultraviolet region (200-400 nm), with a prominent characteristic absorption peak and absorption intensity that decreased exponentially as the wavelength increased into the visible region. CFC-CDs show typical absorption characteristics of carbon-based nanomaterials: a broad absorption peak at 260 nm indicated that the sample contained a UV-absorbing chromophore (Figure 1F), which agrees well with literature reports on π-π* electronic transitions of sp^2^ carbon domains (Das et al., 2022).

One of the most appealing properties of CDs is their photoluminescence, and the luminescence mechanism is derived from the quantum confinement effect, surface state luminescence, or doping effects (Jorns and Pappas, 2021). This study investigated the photophysical properties of the produced CFC-CDs. Fluorescence spectral analysis showed that CFC-CDs emitted blue light under 365 nm UV excitation and appeared yellow in water under natural light (Figure 1G). The spectral parameters were obtained by systematic measurement: λex,max = 351 nm and λem,max = 452 nm. The fluorescence of CDs includes tunability and excitation dependence. For example, the emission can be tuned from blue to near-infrared by adjusting the temperature (Wang et al., 2023). Varying the excitation wavelength shifts the emission peak, as reported for various types of CDs (Li et al., 2015). Based on the fluorescence characterization data, the excitation wavelength response of CFC-CDs was further analyzed. Two typical spectral characteristics were found when excitation scanning was performed at intervals of 20 nm over the range of 300–400 nm (Figure 1H). First, as the excitation wavelength increased (300–400 nm), the emission peak showed a red shift, and the shift range was Δλ = 46 nm. The fluorescence intensity peaked at an excitation wavelength of 340 nm after an initial increase and subsequent decrease. This correlation between excitation wavelength and both emission wavelength and intensity was highly consistent with the photophysical behavior of carbon-based nanomaterials reported in the literature (Pan et al., 2015), which confirmed that CFC-CDs have excitation-wavelength-dependent emission characteristics typical of carbon nanomaterials.

FTIR spectroscopy is a valuable tool for characterizing surface functional groups. At a wavenumber of 3428 cm^-1^, an absorption peak was observed, indicating the stretching of O–H bonds. A broad absorption band indicates the presence of hydrogen bonds within the molecule. Additionally, a peak around 2,920 cm^-1^ signifies the stretching of C–H bonds. The absorption maxima at 1,631 cm^-1^, 1,384 cm^-1^, and 1,097 cm^-1^ corresponded to the stretching of C=O, C–H, and C–O–C groups, respectively (Figure 1I). Spectral analysis revealed the presence of a carbon-based framework in CFC-CDs, featuring oxygen-rich functional groups on the surface and intermolecular hydrogen bonds. The presence of these groups is an important factor for the good water solubility and dispersibility of CFC-CDs, and these groups also improve their stability and cellular uptake efficiency.

XPS was used to characterize the surface functional groups and elemental composition of CFC-CDs. Analysis of the XPS data in Figures 1J–M indicated that the surface of CFC-CDs mainly comprises three elements: C 1s (57.13%), N 1s (4.13%), and O 1s (38.74%). The high-resolution XPS C 1s spectrum was resolved into four characteristic peaks: a C-C/C=C bond at 284.77 eV, a C-N/C-O bond at 286.35 eV (Garcia-Millan et al., 2022), and a C=O/C=N bond at 288.65 eV (Ghosh et al., 2023). Fine spectroscopic analysis of O 1s revealed the presence of this element in two chemical states: a C-O bond at 531.77 eV and a C=O bond at 532.62 eV (Qiu et al., 2023). Analysis of the chemical environment of nitrogen revealed two characteristic binding energies: an N–H bond at 399.92 eV (Hu et al., 2022) and a C–N bond at 401.88 eV (Gao et al., 2022). According to XPS, the surface of CFC-CDs is rich in oxygen- and nitrogen-based functional groups such as hydroxyl, carboxyl, amine, and ether. This finding is consistent with earlier FTIR analyses and strengthens our understanding of the surface chemistry of CFC-CDs.

CFC-CDs exhibited a broad XRD peak. This broad peak suggested that the crystal structure of CFC-CDs was disordered and existed in the form of amorphous carbon. The sharp peak indicated that CFC-CDs had a crystal or near-crystal structure, which resembles the amorphous carbon core produced via the bottom-up approach. Figure 1N shows a diffraction peak at 2θ = 27.31°, and the lattice spacing corresponding to this diffraction peak was approximately 0.210 nm, which is consistent with the lattice spacing observed in the HRTEM image above.

To effectively exclude the interference of small-molecule compounds contained in CFC-CDs with subsequent research, HPLC was used in this study to compare and analyze the components of Crataegi Fructus and CFC-CDs. The chromatogram of the Crataegi Fructus extract showed relatively rich chromatographic peak signals, which may be related to the characteristic flavonoids it contains, such as vitexin and quercetin. It was worth noting that no characteristic peak corresponding to the Crataegi Fructus components was detected in the CFC-CDs solution subjected to high-temperature carbonization and a multi-step purification process, which strongly indicated that the small-molecule compounds contained in Crataegi Fructus could be effectively removed by the optimized preparation process, thus excluding their possible influence on the subsequent experimental results (Figure 1O). Consequently, the aforementioned features support the effective synthesis of CFC-CDs with ultra-small dimensions and high purity.

### 3.2 Biocompatibility of CFC-CDs

Crataegi Fructus, as a common medicinal and edible material, has shown high safety in medical research and clinical use for thousands of years, which ensures the biosafety of materials from the source. However, the biological safety of nanomaterials has attracted widespread attention since their inception and has also become a necessary test item for their application in biotherapy. We assessed their biocompatibility by measuring the hemolysis index across a concentration range of 31.25–1,000 μg/mL. Body weight, liver function, and histological changes in key organs were compared between the control and CFC-CDs groups.

Hemolysis index tests are commonly used to evaluate the biocompatibility of biological materials, and internationally, a material is generally considered biocompatible only when its hemolysis rate is below a certain threshold. When the hemolysis rate is less than 5%, good biocompatibility can be indicated (Fornaguera and Solans, 2017). The biocompatibility of CFC-CDs was assessed by *in vitro* analysis of the hemolysis index at different concentrations (31.25–1,000 μg/mL). The generated CFC-CDs, which exhibited exceptional stability, may facilitate novel uses in biological contexts. The hemolysis rate of CFC-CDs exhibited a positive correlation with concentration within the range of 31.25–1,000 μg/mL, indicating concentration dependence, but the overall hemolysis rate was far below the international standard (5%). The low hemolysis rate indicated that the presence of CFC-CDs was less destructive to red blood cells under physiological conditions, and CFC-CDs showed good blood compatibility, which supports the safety of subsequent *in vivo* studies (Figures 2A,B).

**FIGURE 2:**
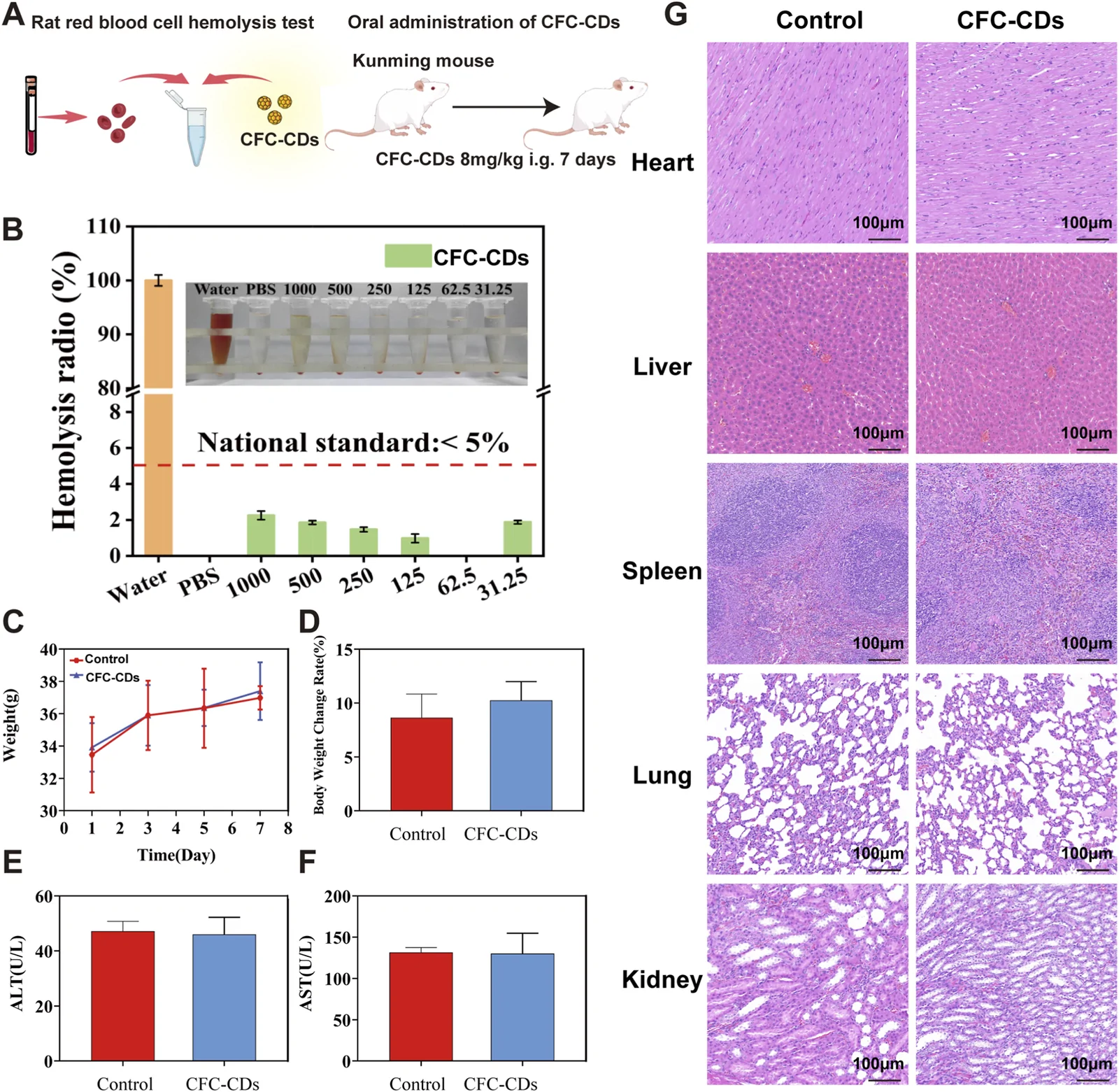
Evaluation of the biocompatibility of CFC-CDs. **(A)** Diagram illustrating the experimental layout for assessing the biocompatibility of CFC-CDs. **(B)** Hemolysis testing of CFC-CDs (31.25–1,000 μg/mL) (n = 6). **(C,D)** Body weight and body weight changes in mice after oral administration of CFC-CDs (n = 6). **(E)** Serum ALT levels. **(F)** Serum AST levels. **(G)** Pathological images of major organs stained with H&E after oral administration of CFC-CDs. Scale bar: 100 μm. Results are expressed as mean ± SD. (^#^P < 0.05, ^##^P < 0.01, ^###^P < 0.001, vs. control).

Body weight serves as a reliable indicator of the growth status of mice. Mouse body weight was documented every 2 days after intragastric administration of CFC-CDs. Over the course of a week, the body weight of mice increased consistently, closely mirroring the growth trajectory of the control group without notable discrepancies. Although the CFC-CDs group showed a slightly faster growth rate than the control group, the difference was statistically insignificant (P > 0.05). CFC-CDs administered by gavage for 7 days had no detrimental impact on the growth of mice (Figures 2C,D).

Nanoparticles may have toxic effects on the liver and kidney after entering the body (Roointan et al., 2025). Therefore, serum ALT and AST levels were measured to assess changes in liver function. Mice given five times the oral dose of CFC-CDs daily for 7 days showed no significant changes in ALT or AST levels compared with the control group (Figures 2E,F). These findings indicated that continuous administration of CFC-CDs at 4–5 times the oral dosage did not significantly affect liver function in mice, demonstrating the good *in vivo* biosafety of CFC-CDs. Since HL requires long-term drug intervention, we evaluated the safety of major organs. The potential toxic effects of oral CFC-CDs on multiple organs in mice were assessed by H&E histopathological staining after 7 days of continuous high-dose administration. Histological examination of the heart, liver, spleen, lungs, and kidneys revealed no inflammation, necrosis, or structural defects (Figure 2G). The above findings indicated that CFC-CDs exhibited good biocompatibility, reflecting the biotherapeutic advantages of nanomaterials and providing sufficient evidence for the safety of subsequent biological experiments.

### 3.3 Impact of CFC-CDs on rats’ hyperlipidemia-induced general condition

Following 7 weeks on a high-fat regimen, the HFD group exhibited marked metabolic abnormalities compared with the control group. Over the course of the study, the HFD group exhibited pronounced physical changes, with animals developing noticeable abdominal fat accumulation from accelerated subcutaneous fat deposition (Figures 3A,B). This progressive weight gain led to a clear obese phenotype, as evidenced by measurable increases in Lee’s index values (P < 0.001) and a marked rise in body mass by the end of the study (P < 0.01) (Figures 3C,E,F). The body weight growth rate reached 70% (P < 0.001), accompanied by a trend of increasing food intake compared with other treatment groups, but not as much as the control group (Figure 3D). This may result from the varying energy composition of the food (Dong P. et al., 2024). After administration of ATV and various doses of CFC-CDs, body weight gain and fat accumulation were substantially reduced. The intervention group exhibited effective control over body weight and fat deposition, resulting in a considerable reduction in Lee’s index and body weight (P < 0.05). Food intake also exhibited a decreasing trend, which may be because CFC-CDs increase energy metabolism and provide sufficient energy for the body, thereby reducing food intake. These phenotypic findings suggest that treatment with CFC-CDs can significantly reverse the obese phenotype resulting from a high-fat diet. Compared with other drug administration groups, the high-dose CFC-CDs group showed greater improvement in the general condition of rats with hyperlipidemia.

**FIGURE 3:**
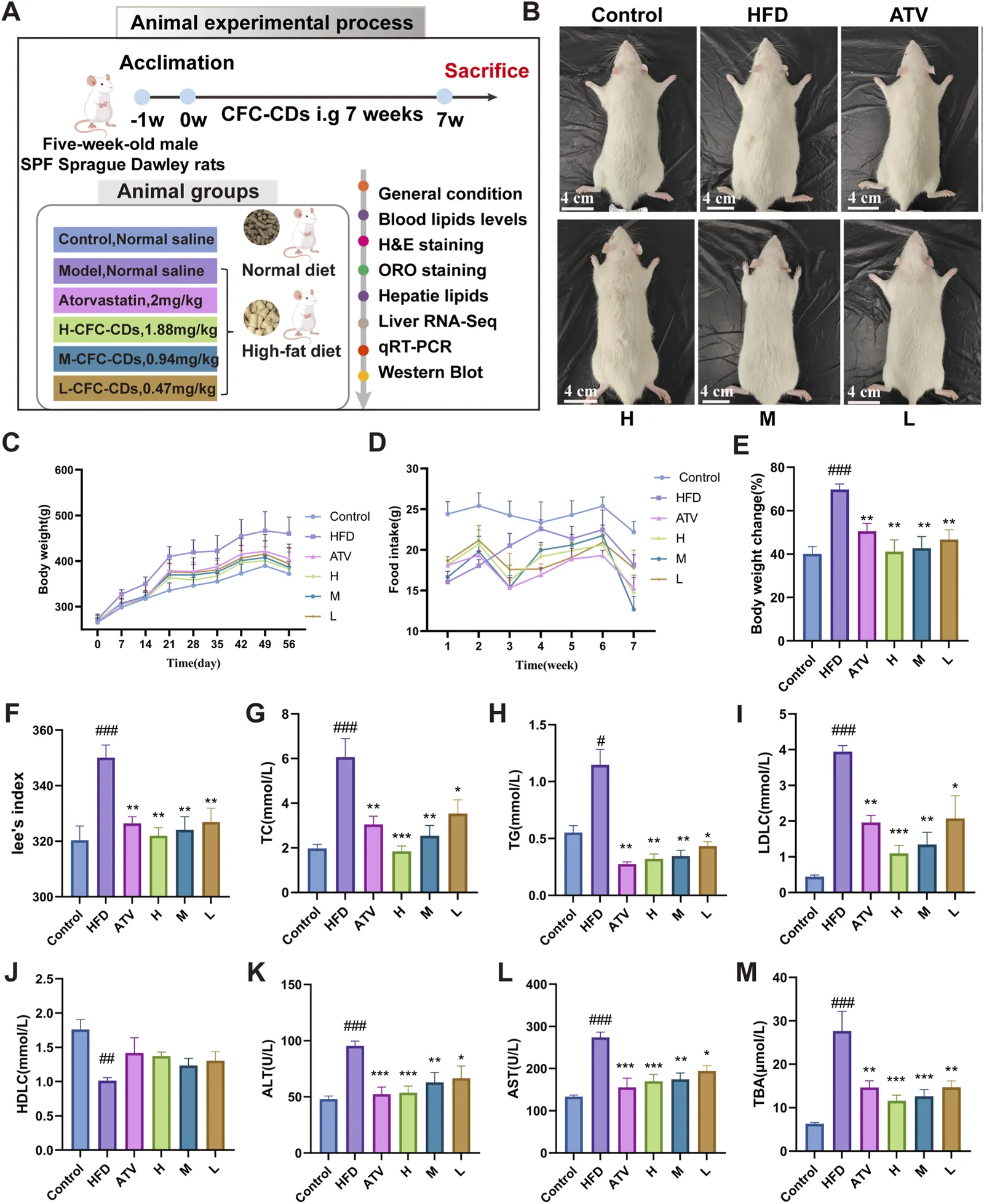
Effects of seven weeks of CFC-CDs intervention on general conditions and serum lipid parameters in rats. **(A)** Experimental grouping and drug administration. **(B)** Representative images of the appearance and body condition of rats in each group. **(C)** Body weight. **(D)** Food intake. **(E)** Body weight change rate. **(F)** Lee’s index. **(G)** Serum TC. **(H)** Serum TG. **(I)** Serum LDL-C. **(J)** Serum HDL-C. **(K)** Serum ALT. **(L)** Serum AST. **(M)** Serum TBA levels (n = 8). Results are expressed as mean ± SD and analyzed using one-way ANOVA. (^#^P < 0.05, ^##^P < 0.01,^###^P < 0.001, vs. control; ^*^P < 0.05, ^**^P < 0.01, ^***^P < 0.001 vs. HFD).

### 3.4 Effects of CFC-CDs on blood lipid levels in hyperlipidemic rats

High-fat feeding significantly affected lipid metabolism homeostasis in animals. In comparison to the serum lipid parameters of the control group, the HFD group exhibited characteristic dyslipidemia: TC, TG, and LDLC (P < 0.05) were considerably increased, and HDLC levels were dramatically diminished (P < 0.01). Following the administration of CFC-CDs, all dosage groups—high, medium, and low—exhibited significant improvements in dyslipidemia status. Compared with the HFD group, the ATV group exhibited significant reductions in TC, TG, and LDLC values, CFC-CDs-H, CFC-CDs-M, and CFC-CDs-L dose groups, with statistically significant differences. Notably, while the ATV group and the different doses of CFC-CDs groups did not significantly reverse HDLC levels, they all showed a trend toward a moderate increase in HDLC levels (Figures 3G–J).

To evaluate the consequences of CFC-CDs on hepatic function and bile acids in hyperlipidemic rats, we measured serum ALT, AST, and TBA levels in each rat group. The HFD group showed significantly higher levels of these markers than the control group (P < 0.001). This might be attributed to lipid buildup in the liver caused by a high-fat diet, aggravating lipotoxicity, which causes rupture of hepatocyte membranes and enzyme release, consequently elevating liver enzyme levels and bile acid concentration in the serum. After treatment with ATV and different doses of CFC-CDs, liver function was significantly restored (P < 0.05) (Figures 3K–M).

### 3.5 Effects of CFC-CDs on hepatic lipid levels in hyperlipidemic rats

In the control group, the liver was dark red, with a smooth surface, a soft texture, and sharp edges. After 7 weeks of high-fat feeding, the rats’ livers became reddish-yellow, the texture became softer and greasier, and the edges became blunt and thick (Figure 4A). This was due to the deposition of excessive energy intake in the liver. Compared with the control group, the HFD group showed a significantly higher liver index, as well as markedly elevated hepatic TC and TG levels. The administration of varying dosages of CFC-CDs dramatically restored hepatic morphology and corrected lipid abnormalities induced by a high-fat diet in a dose-dependent manner. The ATV group also showed significant improvement (P < 0.05). These observations indicate that CFC-CDs may reduce hepatic lipid accumulation by modulating hepatic lipid metabolism (Figures 4B–D).

**FIGURE 4:**
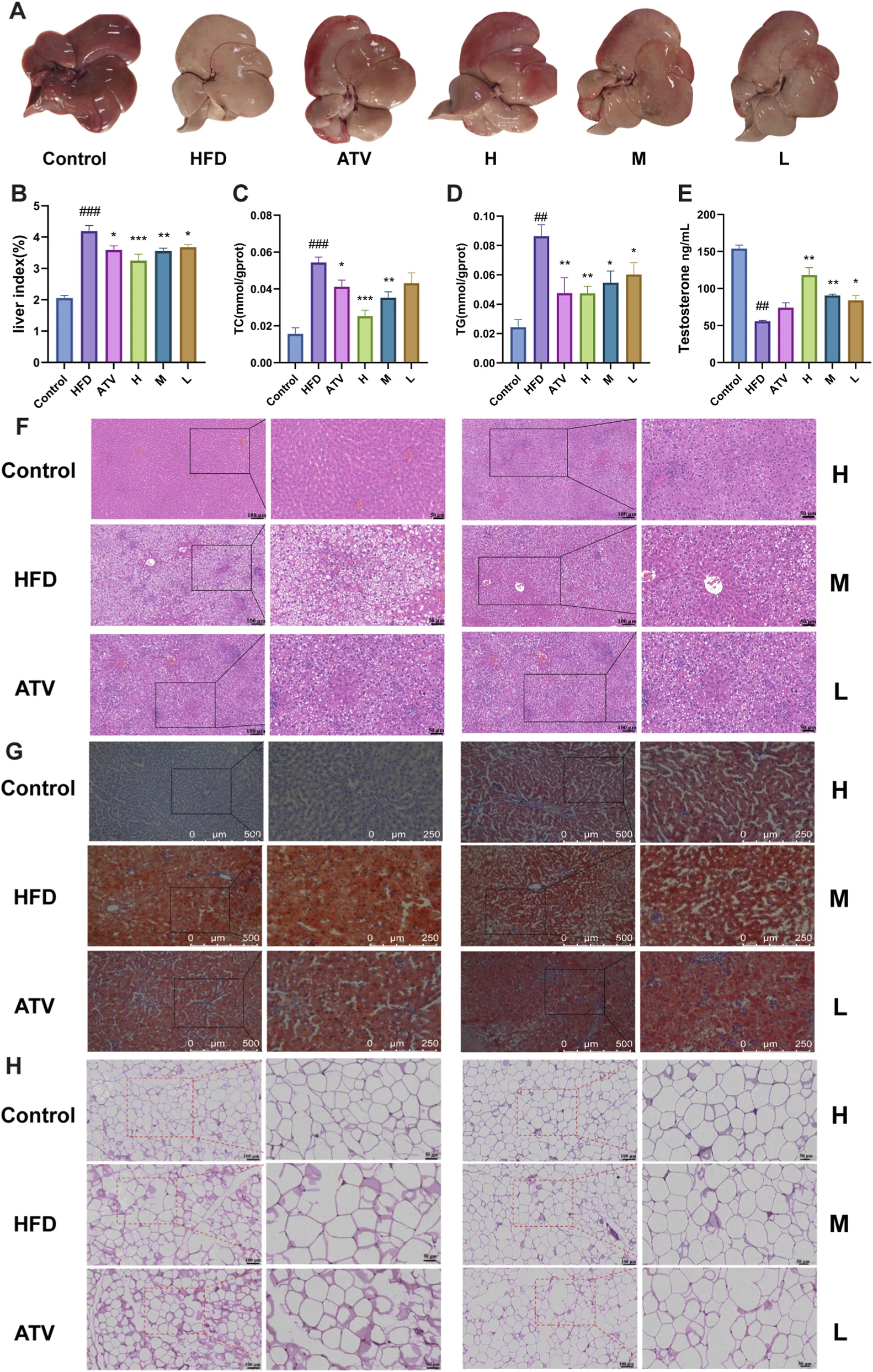
Effects of CFC-CDs administration on the appearance of rat livers and liver lipids. **(A)** Representative images of the hepatic morphology of rats in each group. **(B)** Liver index. **(C,D)** Liver TC and TG levels. **(E)** Serum testosterone concentrations. **(F)** Representative images of H&E histological sections of liver. Scale bar = 100 μm and 50 μm. **(G)** Representative image of Oil Red O staining of rat liver. **(H)** Representative image of H&E staining of rat adipose tissue. Scale bar = 100 μm and 50 μm. Results are expressed as mean ± SD and analyzed using one-way ANOVA. (^#^P < 0.05, ^##^P < 0.01,^###^P < 0.001, vs. control; ^*^P < 0.05, ^**^P < 0.01, ^***^P < 0.001 vs. HFD).

Testosterone is synthesized directly from cholesterol. The consumption of saturated fatty acids and cholesterol in a high-fat diet may enhance testosterone synthesis by supplying additional precursors; however, excessive ingestion might adversely affect testosterone levels. Testosterone levels in the high-fat diet group were markedly lower than those in the control group. After treatment with ATV and various CFC-CD doses, serum testosterone levels exhibited a considerable increase. This may be because, after drug intervention, a portion of the saturated fatty acids and cholesterol was metabolized, thereby reducing their effects on testosterone levels (Figure 4E).

Rapid lipid accumulation with vacuolization is a typical hepatic histopathological finding in rats fed a high-fat diet. HE staining of the liver revealed that fat accumulation caused round or oval clear vacuoles in the cytoplasm of hepatocytes. The surrounding cytoplasm was compressed into a thin ring, hepatocyte volume increased, fat vacuoles of varying sizes appeared in the cytoplasm, the nucleus was displaced to the periphery, and hepatic plates were disorganized in areas of fat accumulation. The normal relationship between the central vein and the portal area was obscured, pseudolobules formed, and pyknosis of hepatocyte nuclei was observed in areas of severe fat accumulation, indicating that the model was successfully established by feeding a high-fat diet for 7 weeks. In the control group, the hepatic tissue structure was distinct, hepatocytes exhibited a polygonal shape, the cytoplasm was uniformly stained red, and no vacuoles, granular particles, or lipid droplets were present. There was no eosinophilic or ballooning degeneration. The nucleus was large and centrally located, the nuclear membrane was smooth and clear, and the chromatin was evenly distributed. The hepatic lobules were centered on the central vein, and the hepatic cells were well-demarcated and radially arranged to form regular hepatic plates (1 cell thick). The hepatic cords and sinusoids were alternately arranged, and the vascular and biliary structures were neither distorted nor compressed. After administration of ATV and CFC-CDs, hepatocyte steatosis was markedly reduced, and hepatocyte morphology was restored (Figure 4F).

In normal liver tissue, hepatocytes usually contain little or no fat. After Oil Red O staining (Figure 4G), only a few scattered small red lipid droplets were visible. The HFD group exhibited extensive Oil Red O-stained lipid droplets, often in clusters, indicating pronounced hepatic lipid accumulation. Following the administration of ATV and CFC-CDs, hepatic lipid deposition was substantially improved. The improvement in hepatic lipid deposition in the high-dose CFC-CD group was greater than that in the ATV group.

Normal adipose tissue predominantly consists of mature adipocytes. The adipocytes were round or polygonal and vacuolated, with a single large lipid droplet in the cytoplasm (resulting from lipid dissolution during processing). The size and shape of the adipocytes were relatively uniform, and the nuclei were flat and round, located at the periphery. However, in the HFD group, the nuclei were squeezed to the periphery and appeared flat and round, and the adipocytes showed a larger outline and volume. The average area of adipocytes increased significantly, the cells were loosely arranged, and vacuole fusion even appeared. After treatment with ATV and CFC-CDs, the adipocytes became smaller and were more compactly arranged (Figure 4H).

### 3.6 Transcriptome sequencing results from rat liver, followed by RT-qPCR and WB for mechanism validation

The Illumina NovaSeq 6000 high-throughput sequencing platform was used to analyze the transcriptome of liver tissue in hyperlipidemic rats (Figure 5A). By comparing gene expression profiles before and after CFC-CD administration, the biological processes and molecular mechanisms of CFC-CDs in HFD rats were further analyzed. Between the control and HFD groups, 3,612 genes were found to be differentially expressed, while 384 genes distinguished the HFD group from the CFC-CD group (Figure 5B). Analysis of differential gene expression in the transcriptome using a histogram revealed striking disparities between the HFD and control groups. The HFD group exhibited substantial alterations in gene expression, with 2,062 genes showing increased expression and another 1,550 showing reduced expression compared with the control group (Figure 5C). Compared with the HFD group, 275 genes were upregulated and 109 downregulated after CFC-CD treatment. These differentially expressed genes are clearly shown in the volcano plot (Figures 5D,E), and this graphical representation allows direct comparison of gene expression patterns after high-fat diet feeding and CFC-CD administration.

**FIGURE 5:**
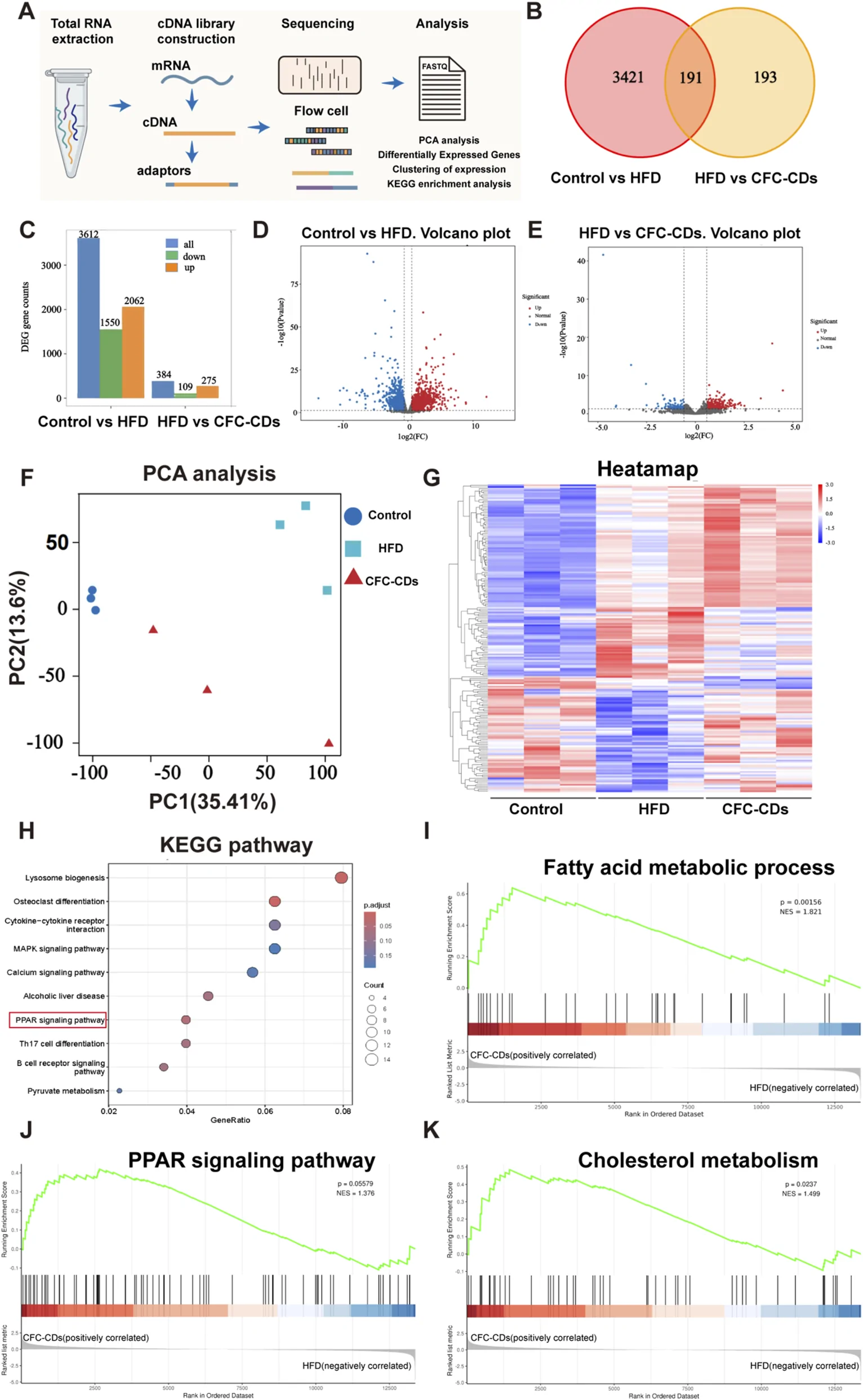
Transcriptomic analysis of rat liver. The animal groups were the control group, the HFD model group, and the high-dose CFC-CDs group. **(A)** Transcriptome sequencing process. **(B)** Venn diagram illustrating the unique and shared differentially expressed genes. **(C)** Statistical bar plot of differentially expressed genes (DEGs). **(D)** Volcano plot illustrating differentially expressed mRNAs between the control and HFD groups. **(E)** Volcano plot illustrating differentially expressed mRNAs between the HFD and CFC-CDs groups. **(F)** Principal component analysis of the distribution of each sample in the three groups and the differences between groups. **(G)** Heatmap of cluster analysis of DEGs in each group of rats. **(H)** KEGG enrichment bubble plot of DEGs. **(I)** GSEA results for genes involved in differences in fatty acid metabolism between the HFD and CFC-CDs groups. **(J)** GSEA results for DEGs in the PPAR signaling pathway between the HFD and CFC-CDs groups. **(K)** GSEA results for DEGs in cholesterol metabolism between the HFD and CFC-CDs groups.

Principal component analysis (PCA) was applied to assess the global variability profile of the transcriptome dataset. This method can effectively evaluate inter-group variability and intra-group consistency through dimensionality reduction, and identify potentially abnormal samples to improve data reliability. As shown in Figure 5F, there is a clear separation along the principal components among the control, HFD, and CFC-CD groups. Although CFC-CD-treated samples showed some degree of within-group biological variation, they showed an overall trajectory from the HFD group toward the control group, consistent with the observed reduction in hepatic lipid deposition, suggesting that CFC-CDs may exert a therapeutic effect by correcting metabolic disorders.

The clustering heatmap shows gene expression patterns across samples under different treatment conditions: control, HFD, and CFC-CDs (Figure 5G). The hues on the heatmap, ranging from blue to red, signify varying degrees of gene expression, with blue denoting low levels and red indicating high levels. A closer look at the heatmap data reveals a notable discrepancy in gene expression profiles across the various treatment groups. The high-fat diet group exhibited dramatic shifts in gene expression relative to the control group. Gene expression profiles were comparable between the CFC-CD group and the control group, suggesting that CFC-CD therapy effectively reversed the high-fat diet-related gene alterations, leading to a lipid-lowering effect.

The KEGG database constructs a complete biological pathway annotation system by analyzing the molecular interaction networks of genes, proteins, and metabolites. Functional genomics of signaling pathways in the database helps explain cellular metabolic regulation at the molecular level and, by comparing pathway activities in different states, reveals how diseases arise and progress. In contrast, the KEGG enrichment bubble plot (Figure 5H) visualizes each pathway as a circle, with the horizontal axis representing the enrichment factor—a metric comparing the proportion of differentially expressed genes in a given pathway with their proportion across all annotated genes. A larger enrichment factor means stronger pathway-specific enrichment. The vertical axis lists pathway names, while circle color denotes the adjusted q-value (corrected for multiple hypothesis testing), with lower values indicating greater reliability. Enrichment analysis revealed that the top 10 core pathways were significantly enriched, particularly the PPAR signaling pathway. This pathway critically regulates hepatic lipid metabolism by modulating genes related to mitochondrial β-oxidation, fatty acid uptake, binding, and transport (Contreras et al., 2013; Han et al., 2025). The findings suggest that the PPAR pathway significantly influences the ability of CFC-CDs to reduce lipid accumulation. The PPAR signaling pathway was significantly upregulated after CFC-CD administration, suggesting that CFC-CDs may exert a critical regulatory effect on lipid metabolism through this pathway.

Gene Set Enrichment Analysis (GSEA) is a classic method in bioinformatics that evaluates the statistically significant enrichment of gene sets in transcriptome data to reveal the intrinsic associations between specific gene sets and biological functional modules. This method can identify differential expression signatures between biological states by comparing global changes in gene expression profiles under different experimental conditions. The comprehensive analysis showed that CFC-CD treatment significantly enhanced fatty acid and cholesterol metabolic pathways (Figures 5I–K), while the PPARα signaling pathway showed an enrichment trend (P > 0.05). In-depth analysis showed that the metabolic reprogramming process was mainly driven by the synergistic effects of key regulatory factors such as PPARα, CPT1A, FABP1, and ACOX1. The expression changes of these differentially expressed genes (DEGs) provided an important molecular basis for analyzing the metabolic regulatory mechanism of CFC-CDs.

We employed RT-qPCR to analyze changes in mRNA expression levels of key components of the PPARα pathway following treatment with CFC-CDs, based on prior transcriptome-based gene predictions. The investigation specifically focused on comparing gene expression profiles before and after the experimental intervention. The transcriptional activities of PPARα and its downstream lipid metabolism-related regulatory genes (CPT1A, FABP1, and ACOX1) were significantly inhibited by a high-fat diet (P < 0.01). Simultaneously, PGC-1α transcriptional coactivator levels were lower than those in the control group (P < 0.05) (Figures 6A–E). Notably, high-dose CFC-CDs reversed this inhibition more effectively than ATV. This result suggests that CFC-CDs can effectively correct HFD-induced dysregulation of lipid metabolic gene expression at the transcriptional level by regulating the PPARα/PGC-1α molecular axis. This regulatory effect was functionally validated by serum lipid metabolism parameters and other therapeutic indicators, and was highly consistent with the significant activation of the PPAR signaling pathway in KEGG enrichment analysis.

**FIGURE 6:**
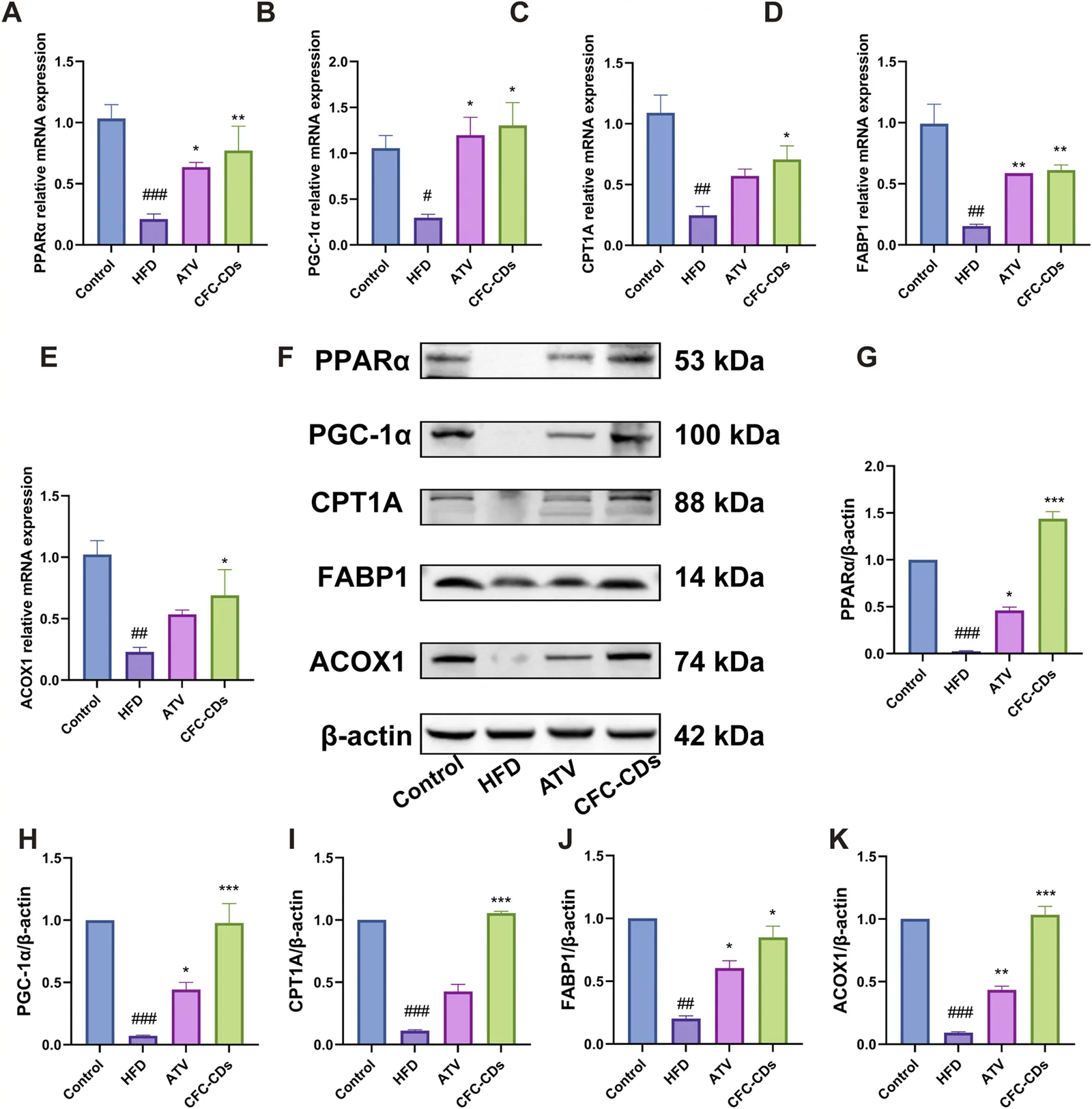
Effects of CFC-CDs on mRNA and protein expression levels of the PPAR signaling pathway in liver tissue. **(A–E)** The mRNA levels of PPARα, PGC-1α, CPT1A, FABP1, and ACOX1. **(F)** Western blot images of liver tissue. **(G–K)** The protein levels of PPARα, PGC-1α, CPT1A, FABP1, and ACOX1. The relative expression levels were calculated after normalizing the gray values of each target protein with the gray values of the internal reference protein. Results are expressed as mean ± SD and analyzed using one-way ANOVA. (^#^ *P* < 0.05, ^##^ *P* < 0.01,^###^ *P* < 0.001, vs. control; ^*^ *P* < 0.05, ^**^ *P* < 0.01, ^***^ *P* < 0.001 vs. HFD).

Our study adopted a multi-dimensional verification strategy. The protein levels of PPARα, CPT-1α, PGC-1α, FABP1, and ACOX1 were evaluated by Western blot. PPARα plays a pivotal role in this metabolic pathway, and after 7 weeks on a high-fat diet, its protein expression was markedly suppressed (P < 0.001). Interestingly, sustained treatment with CFC-CDs dramatically boosted PPARα levels (P < 0.001), effectively counteracting this effect. The primary mechanism by which this protein alleviates hyperlipidemia involves activating downstream target genes, modulating lipid-metabolism gene transcription, and boosting fatty acid β-oxidation in mitochondria and peroxisomes alike. These actions collectively reduce triglyceride accumulation and enhance lipid profiles. Our findings indicate that PPARα activation induced by CFC-CDs markedly elevated the expression levels of CPT1A, FABP1, and ACOX1 (P < 0.01). Furthermore, PGC-1α, a crucial transcriptional coactivator, interacted with PPARα to form a functional complex, thus boosting transcription of fatty acid β-oxidation-related genes. This was accompanied by increased PPARα-related transcription of β-oxidation-related genes (Zhang et al., 2017) and modulation of genes governing fatty acid oxidation. Accordingly, it is plausible to postulate that the cholesterol-lowering efficacy of CFC-CDs is attributable to enhanced mitochondrial β-oxidation. This intricate regulatory network aligns with the earlier observations of improved serum lipid levels and the KEGG pathway data, offering insight into the biochemical mechanisms by which CFC-CDs may restore lipid homeostasis via the PPARα/PGC-1α signaling axis. Notably, compared to ATV, CFC-CDs elicited a stronger activation of PPARα and its downstream genes, with the difference reaching statistical significance. These results validate that a higher dose of CFC-CDs outperforms the ATV group in cholesterol reduction (Figures 6F–K).

### 3.7 Lipid-lowering effects of CFC-CDs on lipid accumulation in HepG2 cells

To determine the optimal oleic acid concentration for inducing lipid accumulation in HepG2 cells, cells were treated with 0, 200, 400, 600, 800, and 1,000 μmol/L OA for 24 h, followed by Oil Red O staining and cell viability assays. Figures 7A,B shows that, relative to the control group, OA treatment elevated intracellular lipid droplet accumulation in a dose-dependent manner. Starting at 200 μmol/L, red lipid droplet staining increased significantly; as the OA concentration increased, lipid droplet accumulation further intensified, and the staining became more pronounced. Cell viability assay results (Figure 7C) showed that treatment with 200 μmol/L and 400 μmol/L OA caused only a mild decrease in cell viability; however, treatment with 600, 800, and 1,000 μmol/L OA significantly reduced cell viability to 80.76%, 80.82%, and 78.32%, respectively, with statistical significance compared with the control group. In summary, OA increases lipid accumulation in HepG2 cells in a dose-dependent manner, yet concentrations over 600 μmol/L cause a notable loss of cell viability. Consequently, 400 μmol/L OA was adopted for subsequent lipid accumulation assays, achieving a balance between significant lipid buildup and adequate cell viability.

**FIGURE 7:**
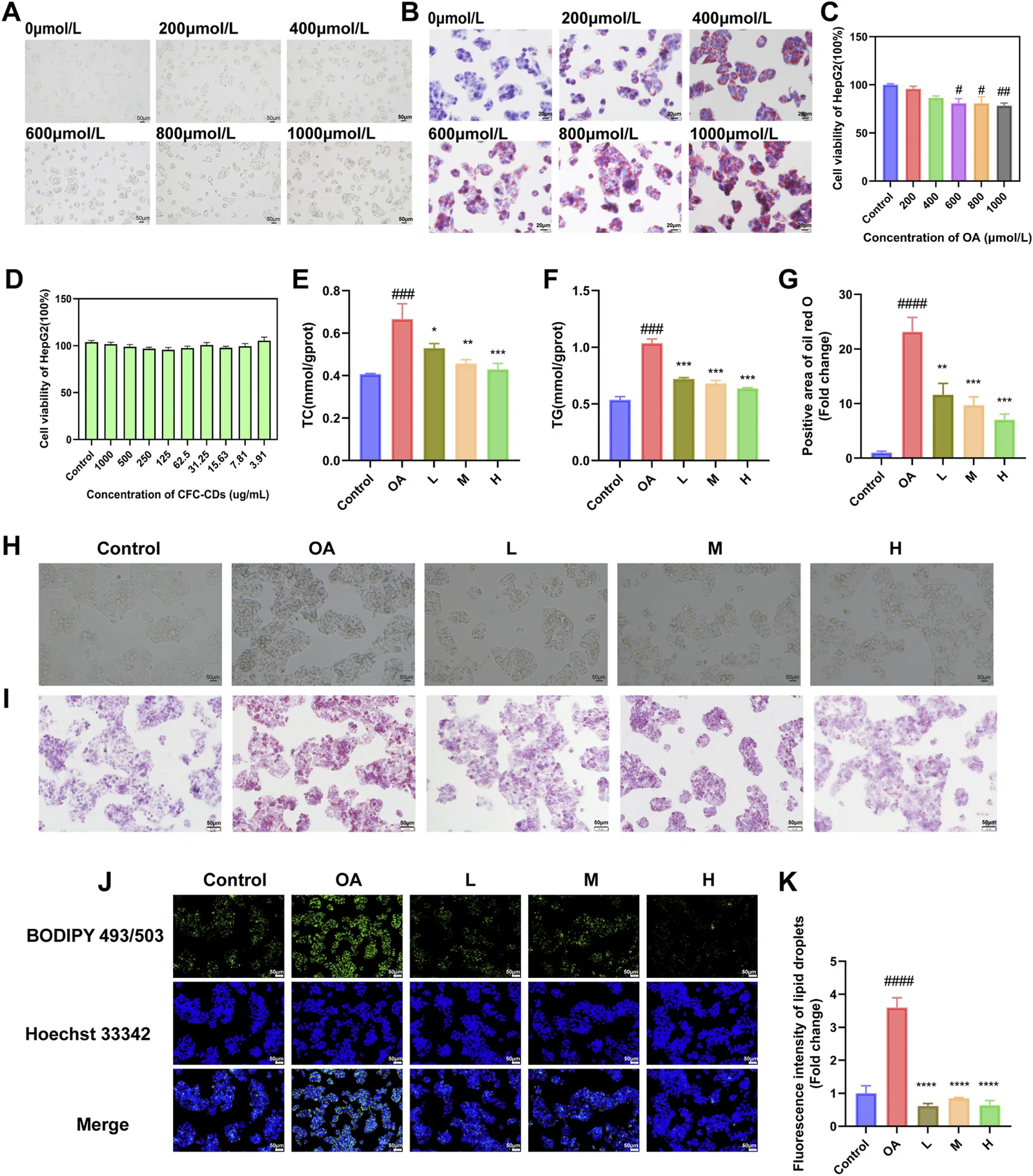
CFC-CDs alleviate OA-induced lipid accumulation in HepG2 cells. **(A,B)** Representative microscopic images and ORO staining of HepG2 cells treated with different concentrations of OA (0, 200, 400, 600, 800, 1,000 μmol/L; incubation time: 24 h). Scale bars = 50 μm and 20 μm. **(C)** Cell viability assessed by CCK-8 assay after treatment of HepG2 cells with different concentrations of OA (0, 200, 400, 600, 800, 1,000 μmol/L). Data are expressed as mean ± SD (n = 4). Compared with the control group (0 μmol/L OA), ^#^p < 0.05, ^##^p < 0.01, ^###^p < 0.001. **(D)** Cell viability of HepG2 cells after 24 h treatment with different concentrations of CFC-CDs (3.91–1,000 μg/mL). **(E)** Total cholesterol (TC) levels (mmol/g protein) in the control group, OA group, and CFC-CDs low (L), medium (M), and high (H) dose groups. **(F)** Triglyceride (TG) levels (mmol/g protein) in each group. **(G)** Relative fold change in the area of ORO-positive staining across groups. **(H,I)** Representative images of cells from each group and ORO staining images. Scale bar = 50 μm. **(J)** BODIPY 493/503 staining (green) labeling lipid droplets, Hoechst 33342 staining (blue) labeling cell nuclei, and a composite image of both. **(K)** Quantitative analysis of lipid droplet fluorescence intensity. Results are expressed as mean ± SD and analyzed using one-way ANOVA. (^#^P < 0.05, ^##^P < 0.01,^###^P < 0.001, vs. control; ^*^P < 0.05, ^**^P < 0.01, ^***^P < 0.001 vs. OA).

An OA-induced lipid accumulation model in HepG2 cells was established to study the lipid-lowering effect of CFC-CDs; the cells were then treated with different concentrations of CFC-CDs. The cytotoxicity of CFC-CDs was evaluated using the CCK-8 assay. As shown in Figure 7D, after 24 h of treatment with CFC-CDs at various concentrations (3.91–1,000 μg/mL), cell viability remained above 90% across the entire concentration range, indicating no significant cytotoxicity. This provides a preliminary understanding of the biosafety of CFC-CDs at the cellular level and provides a positive basis for investigating the complex biological behavior of CFC-CDs, such as their interactions with molecules and proteins within hepatocytes. Therefore, three concentrations (low: 125 μg/mL, medium: 250 μg/mL, high: 500 μg/mL) were chosen for subsequent lipid-lowering activity assays.

As illustrated in Figures 7E,F, OA markedly increased intracellular TC and TG compared with the control group, while CFC-CDs (L, M, H) lowered them to varying degrees compared with the OA group, with the reduction being particularly pronounced in the H group; these differences were statistically significant. This indicates that all CFC-CD dose groups inhibited the excessive accumulation of TC and TG, and this effect exhibited a certain degree of concentration dependence. The OA group exhibited marked lipid accumulation, whereas all CFC-CD dose groups effectively reduced intracellular lipid accumulation and restored the cells to a normal state (Figure 7H). The ORO staining positive rate (Figures 7G,I) reflects the intracellular neutral lipid content. In the control group, the cell contours were clear, with very few red lipid droplets; in the OA model group, obvious red lipid droplets were visible around the cell nuclei, with some droplets fusing into clusters; in the CFC-CD treatment groups (L, M, H), both the number of lipid droplets and the intensity of staining gradually decreased as the concentration increased, indicating that CFC-CDs show excellent lipid-lowering efficacy. To evaluate the effect of CFC-CDs on lipid droplet formation, lipid droplets were stained with BODIPY 493/503 (green fluorescence) and nuclei were stained with Hoechst 33342 (blue fluorescence). As shown in Figure 7J, the OA group exhibited intense and dense green fluorescence, whereas green fluorescence intensity was significantly reduced after CFC-CD treatment. Quantitative analysis of lipid droplet fluorescence intensity (Figure 7K) indicated that, compared with the control group, fluorescence intensity was significantly increased in the OA group; fluorescence intensity decreased progressively in the L, M, and H groups. These results collectively confirm that CFC-CDs can inhibit OA-induced lipid accumulation in HepG2 cells in a concentration-dependent manner.

### 3.8 CFC-CDs reduce OA-triggered lipid buildup in HepG2 cells by activating the PPARα pathway

Based on animal transcriptome sequencing results, the PPARα signaling pathway was identified as a potential key pathway. To validate the role of this pathway *in vitro*, blocking experiments were conducted using the PPARα-specific inhibitor GW6471, and the accumulation of intracellular lipid droplets as well as the expression levels of key downstream proteins of the PPARα pathway were assessed. As shown in Figures 8A,B, following ORO staining, the model group exhibited extensive red staining, while the CFC-CD group showed a reduced number of lipid droplets. The PPARα inhibitor group exhibited a significantly larger red-stained area compared with the OA + H group, demonstrating that GW6471 partly reversed the CFC-CD-mediated inhibition of lipid droplet accumulation.

**FIGURE 8:**
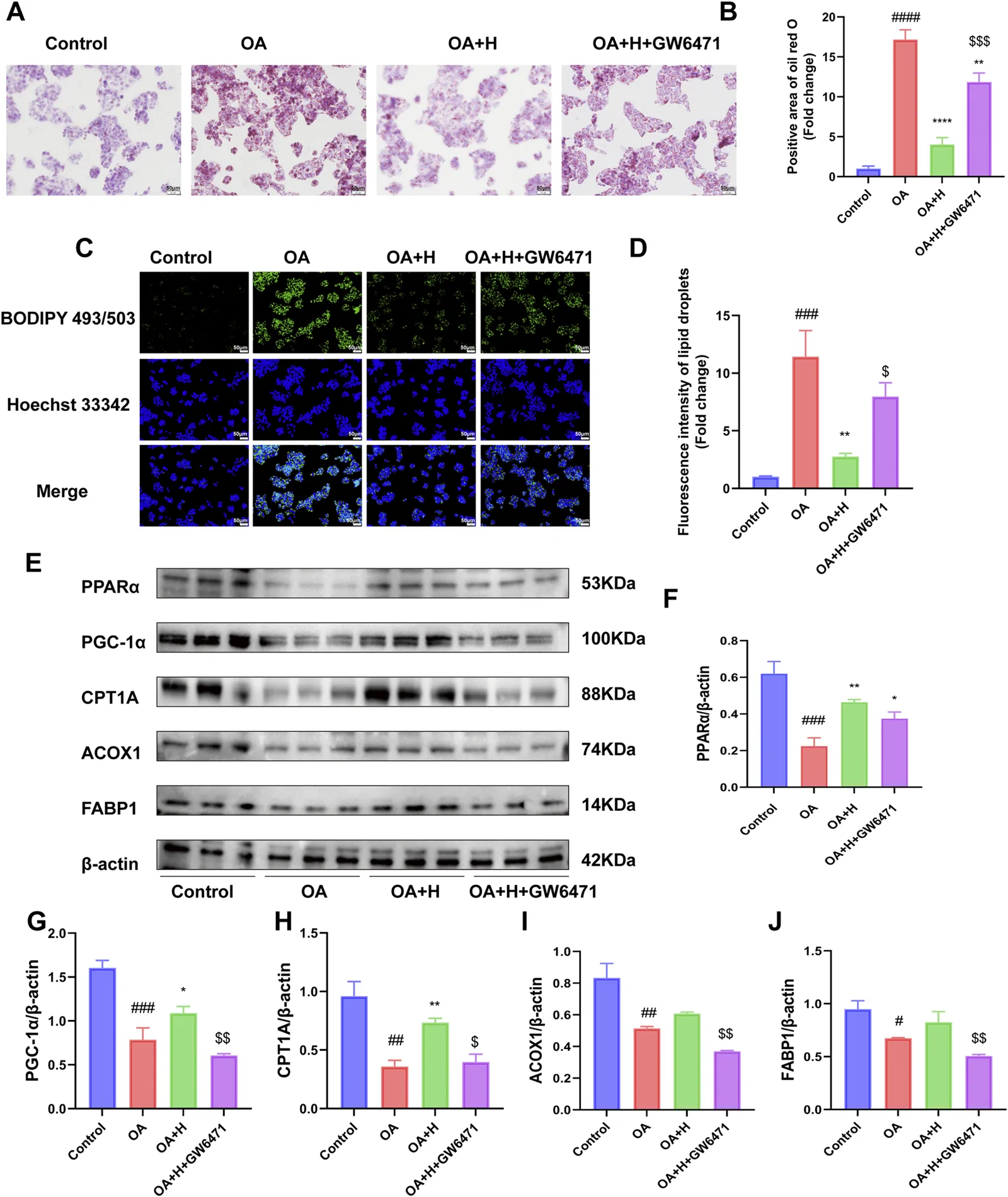
CFC-CDs alleviate OA-induced lipid accumulation in HepG2 cells by activating the PPARα signaling pathway. **(A,B)** Representative images of HepG2 cells stained with ORO and the relative fold change in ORO-positive area for each group: control, OA, OA + H, and OA + H + GW6471. **(C)** Representative fluorescence images of HepG2 cells from each group stained with BODIPY 493/503 (green, marking lipid droplets) and Hoechst 33342 (blue, marking cell nuclei). Scale bar = 50 μm. **(D)** Quantitative analysis of lipid droplet fluorescence intensity, expressed as the fold change relative to the control group. **(E–J)** Western blot analysis of protein expression levels of PPARα, PGC-1α, CPT1A, ACOX1, FABP1, and the loading control β-actin in cells from each group, along with quantitative analysis of the band intensities representing the relative expression levels of each protein. Results are expressed as mean ± SD and analyzed using one-way ANOVA. (^#^P < 0.05, ^##^P < 0.01,^###^P < 0.001, vs. control; ^*^P < 0.05, ^**^P < 0.01, ^***^P < 0.001 vs. OA; ^$^P < 0.05, ^$$^P < 0.01, ^$$$^P < 0.001 vs. OA + H).

To observe the fluorescence intensity of lipid droplets in each group, we subsequently labeled droplets with BODIPY 493/503 (green; Figures 8C,D) and stained nuclei with Hoechst 33342 (blue). The control group exhibited faint green fluorescence and very few lipid droplets. Compared with the control group, OA caused a roughly 12-fold increase in green fluorescence, demonstrating successful induction of extensive lipid droplet accumulation. Green fluorescence was significantly lower in the OA + H group than in the OA group, with fluorescence intensity decreasing by approximately 60%, confirming that CFC-CDs can effectively reduce lipid droplet formation. Compared with the OA + H group, the OA + H + GW6471 group exhibited significantly increased green fluorescence, with an intensity approximately 2.8 times higher, suggesting that the PPARα inhibitor can reverse the lipid-lowering effect of CFC-CDs. These results indicate that the lipid-lowering effect of CFC-CDs may depend on the activation of the PPARα pathway.

We used Western blot to detect the protein expression levels of PPARα, PGC1α, CPT1A, FABP1, and ACOX1. As illustrated in Figures 8E–J, compared with the control group, PPARα expression was downregulated in the OA group (P < 0.001). PPARα expression in the OA + H group was significantly higher than that in the OA group (P < 0.01), whereas the addition of GW6471 partially suppressed PPARα expression compared with the OA + H group. The trend in PGC1α expression was consistent with that of PPARα. PGC1α was upregulated in the OA + H group compared with the OA group, and its expression decreased after GW6471 treatment. As a key fatty acid transport enzyme downstream of PPARα, CPT1A showed markedly higher expression in the OA + H group than in the OA group (P < 0.01); the GW6471 inhibitor effectively blocked this upregulation. ACOX1 exhibited expression changes similar to those of CPT1A: expression levels increased in the OA + H group but declined after GW6471 administration. FABP1 expression was elevated in the OA + H group and returned to baseline levels after GW6471 treatment. These protein expression results collectively indicate that CFC-CDs promote fatty acid catabolism by upregulating the expression of PPARα and its downstream fatty acid oxidation-related proteins (PGC1α, CPT1A, ACOX1, and FABP1), thereby alleviating OA-induced lipid accumulation; this effect was significantly reversed by the PPARα inhibitor GW6471.

### 3.9 Immunofluorescence validation of CFC-CDs promoting fatty acid oxidation via activation of the PPARα signaling pathway

Immunofluorescence staining further validated the cellular activation of the PPARα signaling pathway. PPARα is the core transcription factor responsible for lipid-lowering functions, while PGC-1α is its key co-activator. As shown by the immunofluorescence images of PPARα and PGC-1α (Figures 9A,B), the control group exhibited weak fluorescent signals for PPARα and PGC-1α (green), primarily distributed in the nucleus and perinuclear regions. Compared with the control group, the fluorescence intensities of both PPARα and PGC-1α were reduced in the OA group, suggesting that OA-induced lipid accumulation is accompanied by inhibition of the PPARα signaling pathway. In the OA + H group, the fluorescence intensities of PPARα and PGC-1α were significantly increased, indicating that CFC-CDs upregulated the expression of both. Compared with the OA + H group, the fluorescence intensities of PPARα and PGC-1α in the OA + H + GW6471 group were both significantly reduced, approaching the levels of the OA model group, confirming that GW6471 effectively blocked the CFC-CD-induced upregulation of PPARα and PGC-1α. Quantitative analysis (Figures 9F,G) further supported these findings: the immunofluorescence intensity in the OA + H group was notably higher than that in the OA group (P < 0.05), while the intensity decreased significantly after the addition of GW6471 (P < 0.05).

**FIGURE 9:**
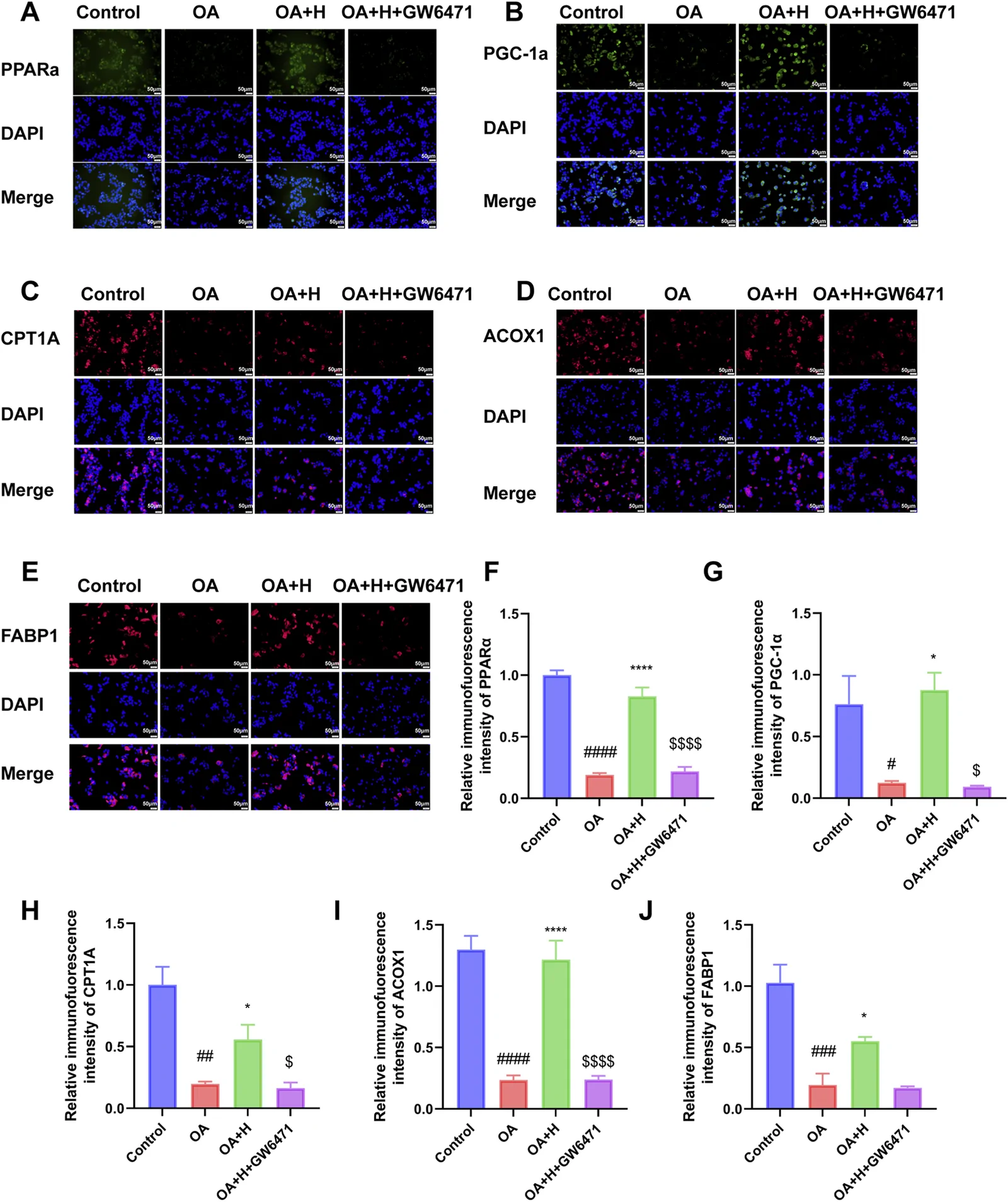
CFC-CDs regulate the nuclear localization and expression of fatty acid oxidation-related proteins by activating the PPARα signaling pathway. **(A–E)** Representative immunofluorescence images of PPARα (green), PGC-1α (green), CPT1A (red), ACOX1 (red), FABP1 (red), and DAPI (blue) in HepG2 cells from each group, along with merged images. Scale bar = 50 μm. **(F–J)** Relative quantitative analysis of PPARα, PGC-1α, CPT1A, ACOX1, and FABP1 fluorescence intensity, respectively. The groups were control, OA, OA + H, and OA + H + GW6471. Results are expressed as mean ± SD and analyzed using one-way ANOVA. (^#^P < 0.05, ^##^P < 0.01,^###^P < 0.001, vs. control; ^*^P < 0.05, ^**^P < 0.01, ^***^P < 0.001 vs. OA; ^$^P < 0.05, ^$$^P < 0.01, ^$$$^P < 0.001 vs. OA + H).

CPT1A functions as the rate-limiting enzyme in fatty acid oxidation; it is situated on the outer mitochondrial membrane and facilitates the import of fatty acids into mitochondria (Tang et al., 2025). In the OA group, CPT1A fluorescence intensity was lower than in the control group; in the OA + H group, intensity was significantly elevated, with an increase in the number and brightness of fluorescent particles, whereas in the OA + H + GW6471 group, fluorescence intensity decreased, approaching the levels observed in the OA group (Figures 9C,H). ACOX1 acts as a critical rate-limiting enzyme in peroxisomal β-oxidation of fatty acids, catalyzing the initial step in the breakdown of long-chain and branched-chain fatty acids. It is localized to peroxisomes and exhibits a punctate distribution in the cytoplasm. Consistent with the trend observed for CPT1A, fluorescence intensity was markedly higher in the OA + H group compared with the OA group (P < 0.05), and intensity decreased after GW6471 treatment (Figures 9D,I), suggesting that this upregulation may be PPARα-dependent. FABP1 is involved in the uptake, cytoplasmic transport, and nuclear distribution of long-chain fatty acids (LCFAs), delivering them to metabolic sites or nuclear receptors; as a fatty acid-binding protein, it is diffusely distributed in the cytoplasm. Fluorescence intensity in the OA + H group was significantly higher than that in the OA group, and GW6471 reversed this effect (Figures 9E,J). The results of immunofluorescence staining were highly consistent with those of Western blot analysis, indicating that treatment with CFC-CDs significantly enhanced the expression of PPARα and its downstream target proteins (PGC-1α, CPT1A, ACOX1, and FABP1), whereas the PPARα-specific inhibitor GW6471 blocked this effect. This further confirms, based on cellular localization and protein expression levels, that CFC-CDs ameliorate OA-induced lipid accumulation by activating the PPARα signaling pathway and upregulating the expression of fatty acid oxidation-related proteins.

## 4 Discussion

Since their initial discovery and description by Scrivens in 2004, carbon dots (CDs)-a new type of brightly luminescent, zero-dimensional carbon nanomaterials-have quickly captured the interest of scientists thanks to their outstanding properties (Xue et al., 2023). This research reveals that CFC-CDs, synthesized through green chemistry, not only retain the lipid-lowering properties of the original herbal material but also exhibit the unique biological advantages of nanomaterials. CFC-CDs likely exert lipid-lowering effects through the integrated properties of their unique nanostructure, surface chemistry, and physical characteristics. Thanks to their minute nano-size and reduced hydrodynamic radius, they are likely to slip through cell membranes more effortlessly, leading to a broader intracellular dispersion. This allows for more efficient interactions with targets such as enzymes or receptors involved in lipid metabolism (Zhang H. et al., 2025; Menichetti et al., 2024). High-temperature carbonization has been shown to change the drug’s properties, which could extend the range of applications for CFC-CDs. Raw Crataegi Fructus may cause excessive gastric acid secretion, pain, calculi, and increased risk of miscarriage (Liu H. et al., 2024; Zhang et al., 2022), while CFC-CDs, as a novel biomass-derived carbonaceous nanomaterial, have significant advantages in reducing acidity, astringency, and gastrointestinal irritation. As “carrier-free” carbon dots derived from edible herbs, CFC-CDs demonstrate unique advantages in nanotherapy for lipid metabolism-related diseases. Compared to anti-obesity nanonutritional formulations that rely on carriers such as liposomes and micelles to encapsulate natural active ingredients (Ni et al., 2025), CFC-CDs require no artificial excipients and inherently possess good aqueous dispersibility and physiological stability. Compared to peptide-modified atherosclerosis-targeted nanoformulations (Liu X. et al., 2024)—which, although highly targeted, are complex to synthesize, prone to hydrolysis, and costly—CFC-CDs, on the other hand, are prepared through a one-step pyrolysis of food-grade raw materials; they have low toxicity and exhibit excellent lipid-lowering efficacy. They can simultaneously restore hepatic metabolic function and reduce TC, TG, and LDL-C upstream, thereby blocking the atherosclerosis-inducing cascade reaction at its source, making them feasible for clinical treatment.

Hyperlipidemia has become an increasingly serious public health problem. It is not only an increase in blood lipid levels, but also a disruption in hepatic lipid metabolic homeostasis. In this pathological state, the liver mistakenly reprograms from a “fat-burning mode” to a “fat synthesis and accumulation mode”. Through excessive intake of saturated fatty acids and cholesterol, a high-fat diet upregulates lipid synthesis genes such as SCD1 and inhibits fatty acid oxidation genes such as PPARα and CPT1A, leading to hepatocyte fat deposition, inducing lipotoxicity, and ultimately resulting in hepatocyte membrane rupture and liver enzyme release (Oosterveer et al., 2009). The liver, functioning as the primary control center for lipid metabolism, is pivotal in orchestrating the movement, creation, storage, and breakdown of lipids, all in coordination with fluctuations in nutrient availability and energy needs (Li et al., 2025). RNA sequencing was employed to investigate gene expression in liver cells. GSEA showed that the PPAR signaling pathway was inhibited and cholesterol metabolism was decreased in the high-fat diet group, while CFC-CDs treatment induced activation of the PPAR signaling pathway, and genes involved in cholesterol metabolism and fatty acid metabolism were significantly upregulated. Therefore, this study focused on the molecular regulatory network mediated by the PPAR signaling pathway to elucidate the biological effects and molecular mechanisms of CFC-CD uptake *in vivo*.

Peroxisome proliferator-activated receptors (PPARs) are members of the nuclear receptor superfamily and regulate genes associated with cell differentiation. They are critically involved in the regulation of lipid, carbohydrate, and energy homeostasis. Three PPAR subtypes-α, β, and γ-have been found in mammals. Each subtype shows distinct tissue distribution patterns, with PPARα serving as the primary subtype in the liver (Pawlak et al., 2015). PPARα is broadly expressed in tissues such as the heart, liver, lung, kidney, intestines, and skeletal muscle. Through heterodimerization with the retinoid X receptor (RXR), PPARα controls gene transcription and consequently contributes to multiple physiological functions, including lipid metabolism, inflammation, and cell proliferation. In the absence of specific ligands, the PPARα/RXR heterodimer binds to PPREs (Peroxisome Proliferator Response Elements) located in the promoter, enhancer, or intron regions of target genes and recruits co-repressors such as NCoR1 (Nuclear Receptor Corepressor 1) to inhibit the transcription of PPARα target genes (Kang and Fan, 2020). Once a specific ligand attaches, nuclear co-repressors are liberated, and co-activators-among them PGC-1α (peroxisome proliferator-activated receptor-γ coactivator-1α) are subsequently recruited (Nakamura et al., 2014). Repression is relieved through histone acetylase (HAT) activity, allowing PPARα target genes involved in hepatic lipid and glucose metabolism to be expressed. These target genes encode fatty acid transporters (e.g., FAT/CD36 and L-FABP) as well as rate-limiting enzymes of peroxisomal β-oxidation, including ACOX1 and EHHADH, which are predominantly expressed in rodents. Our findings demonstrate that CFC-CDs significantly upregulate PPARα expression in the liver. Once activated, PPARα acts as a “metabolic commander” and exerts coordinated regulation of a series of genes involved in fatty acid transport and oxidation by binding to PPREs in the promoter regions of target genes (Fougerat et al., 2024; Pawlak et al., 2015). The activation of PPARα by CFC-CDs signifies a restructured metabolic logic in the liver—from the storage of excess lipids to efficient fatty acid utilization. CPT1A acts as the key rate-limiting enzyme responsible for shuttling long-chain fatty acids into the mitochondria, enabling their β-oxidation. CFC-CDs can significantly induce CPT1A expression, and the upregulated CPT1A can convert excess lipids into energy (ATP). Enhancing the oxidative decomposition of fatty acids in this manner is an ideal strategy for treating hyperlipidemia (Figure 10).

**FIGURE 10:**
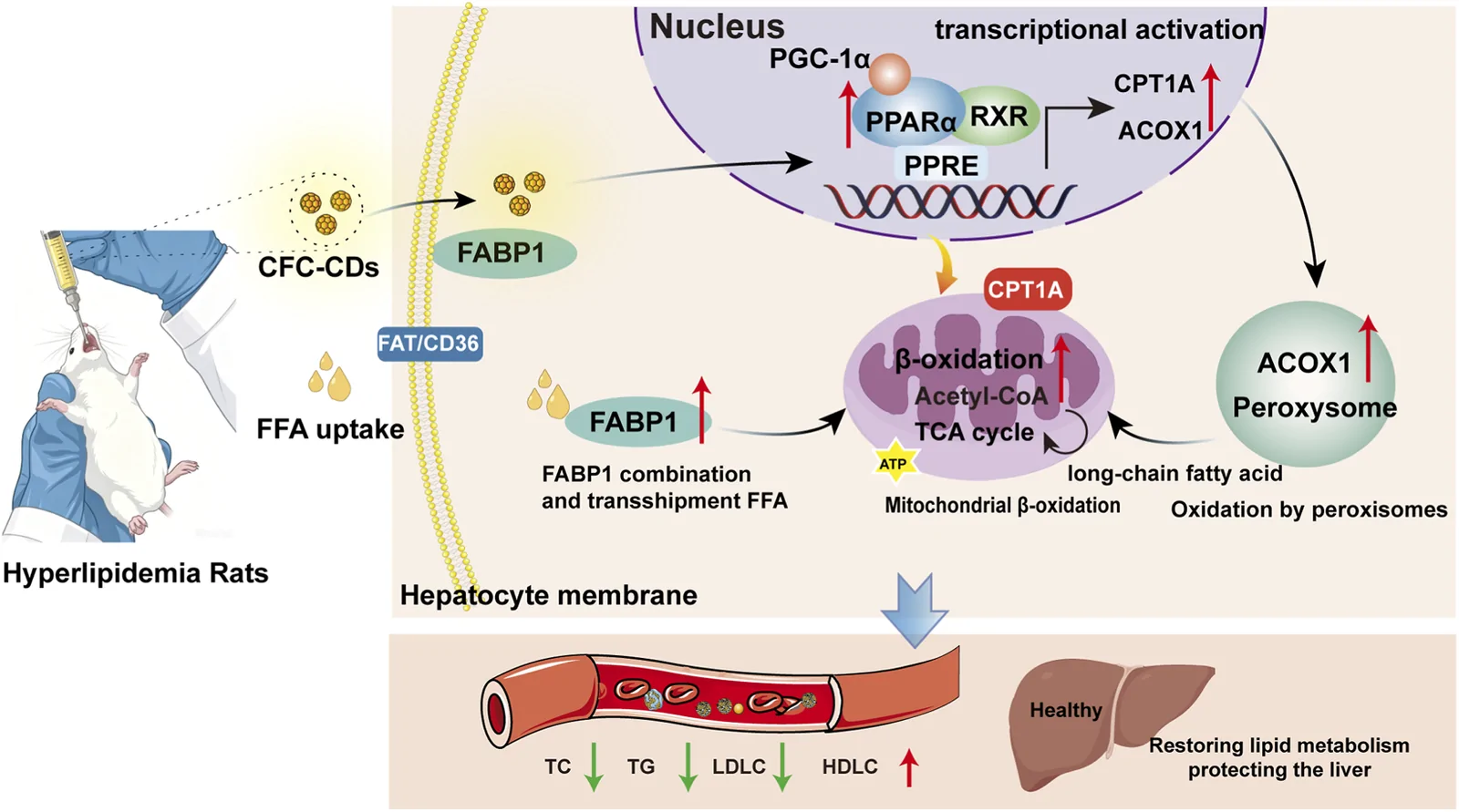
Potential molecular mechanism of CFC-CDs in the treatment of HL.

Although this study preliminarily demonstrated the potential of CFC-CDs in improving lipid metabolism, certain limitations remain. The specific molecular mechanism by which CFC-CDs trigger the upregulation of PPARα remains unclear. The study observed an increase in PPARα expression but did not elucidate how CFC-CDs enter cells and activate this receptor.It remains unclear whether this is achieved through membrane receptor-mediated signaling or through the direct action of carbon dots entering the cell nucleus. The current work is limited to short-term rodent models and isolated cells. It has not yet addressed aspects such as large-animal evaluation, long-term *in vivo* accumulation toxicity, pharmacokinetics in target organs, and *in vivo* degradation and clearance kinetics. Consequently, there remains a long road ahead in preclinical safety and efficacy evaluation before actual clinical application can be achieved.

## 5 Conclusion

In this study, CFC-CDs were prepared via high-temperature pyrolysis and subjected to detailed characterization. The results indicated that CFC-CDs were rich in functional groups such as OH, C=O, and C=N and possessed good biosafety. They demonstrated excellent lipid-lowering activity in both animal and cellular experiments. Transcriptomic sequencing revealed that CFC-CDs primarily alleviated lipid deposition by enhancing fatty acid and cholesterol metabolism through the PPAR signaling pathway, thereby alleviating hyperlipidemia. Taken together, these results provide scientific validation for the use of CFC-CDs against chronic metabolic conditions and suggest a promising transition from traditional dual-function food-drug systems to novel nanomedicine formulations. These findings offer valuable insights into drug development for metabolic diseases by combining traditional wisdom with modern technology.

## Data Availability

The data presented in the study are publicly available and have been deposited in the Zenodo repository. DOI: 10.5281/zenodo.23371052.
