# Supplementary material for: Green edible crataegi fructus-derived carbon dots ameliorate hyperlipidemia via the PPARα signaling pathway
Source: Front Pharmacol. 2026 Oct 16;17:1985522. doi: 10.3389/fphar.2026.1985522 (PMC13720364; doi:10.3389/fphar.2026.1985522)

Fig.6F

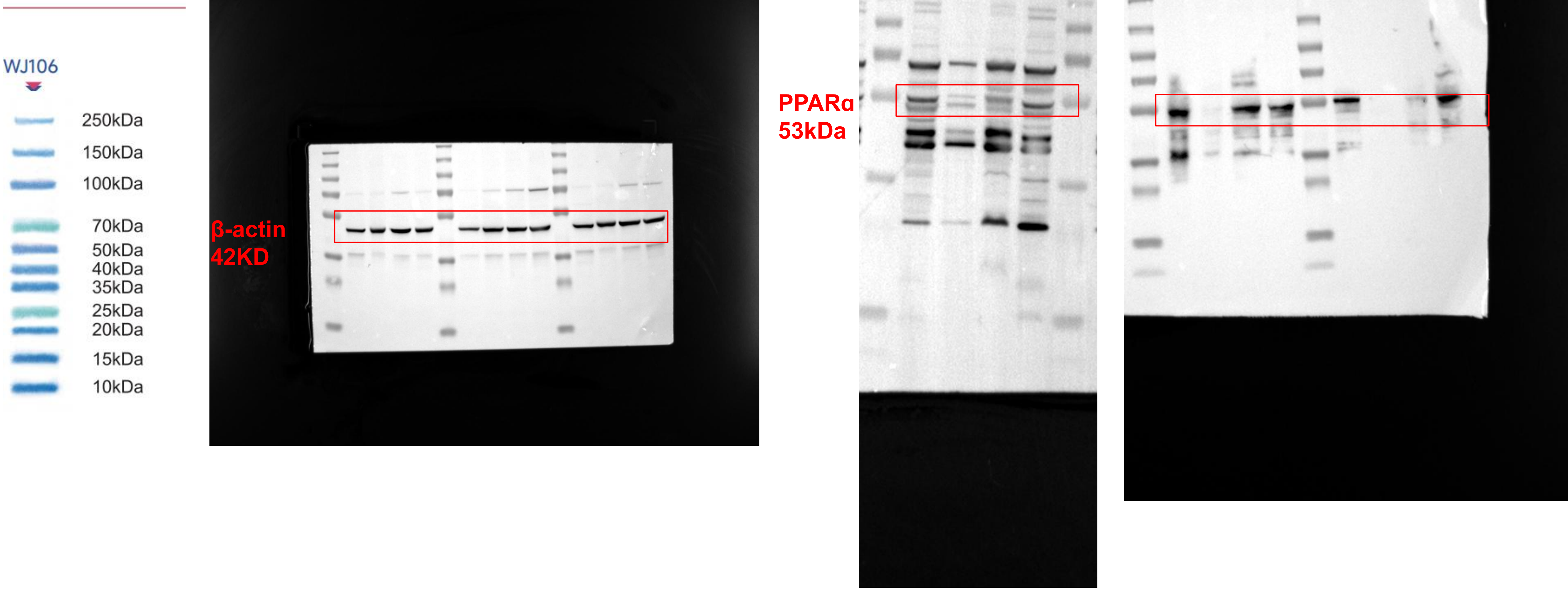

WJ106
250kDa
150kDa
100kDa
70kDa
50kDa
40kDa
35kDa
25kDa
20kDa
15kDa
10kDa
β-actin
42KD
PPARα
53kDa

**Fig.6F**

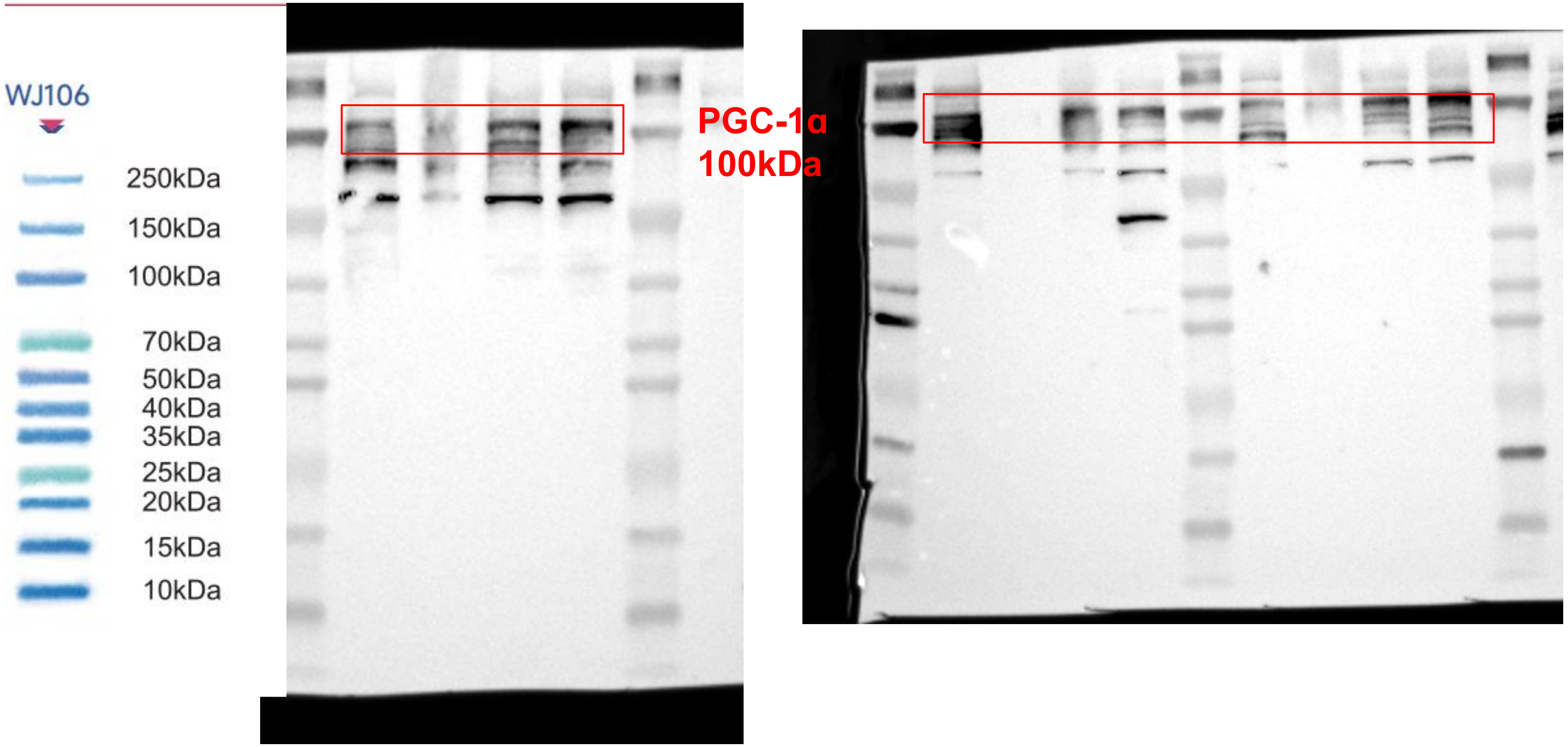
WJ106
250kDa
150kDa
100kDa
70kDa
50kDa
40kDa
35kDa
25kDa
20kDa
15kDa
10kDa
PGC-1α
100kDa

Fig.6F

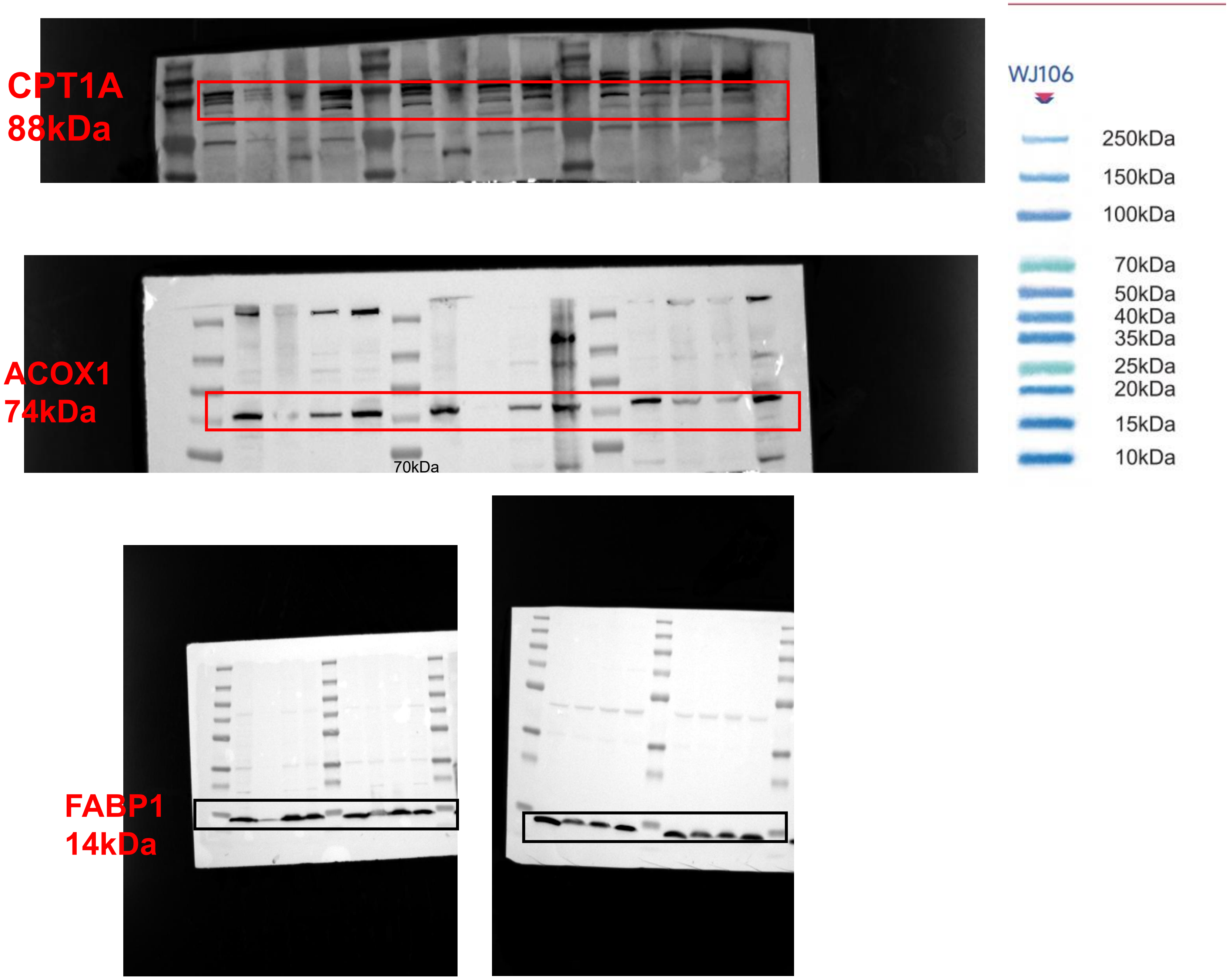

CPT1A
88kDa
ACOX1
74kDa
70kDa
FABP1
14kDa
WJ106
250kDa
150kDa
100kDa
70kDa
50kDa
40kDa
35kDa
25kDa
20kDa
15kDa
10kDa

**Fig.8E**

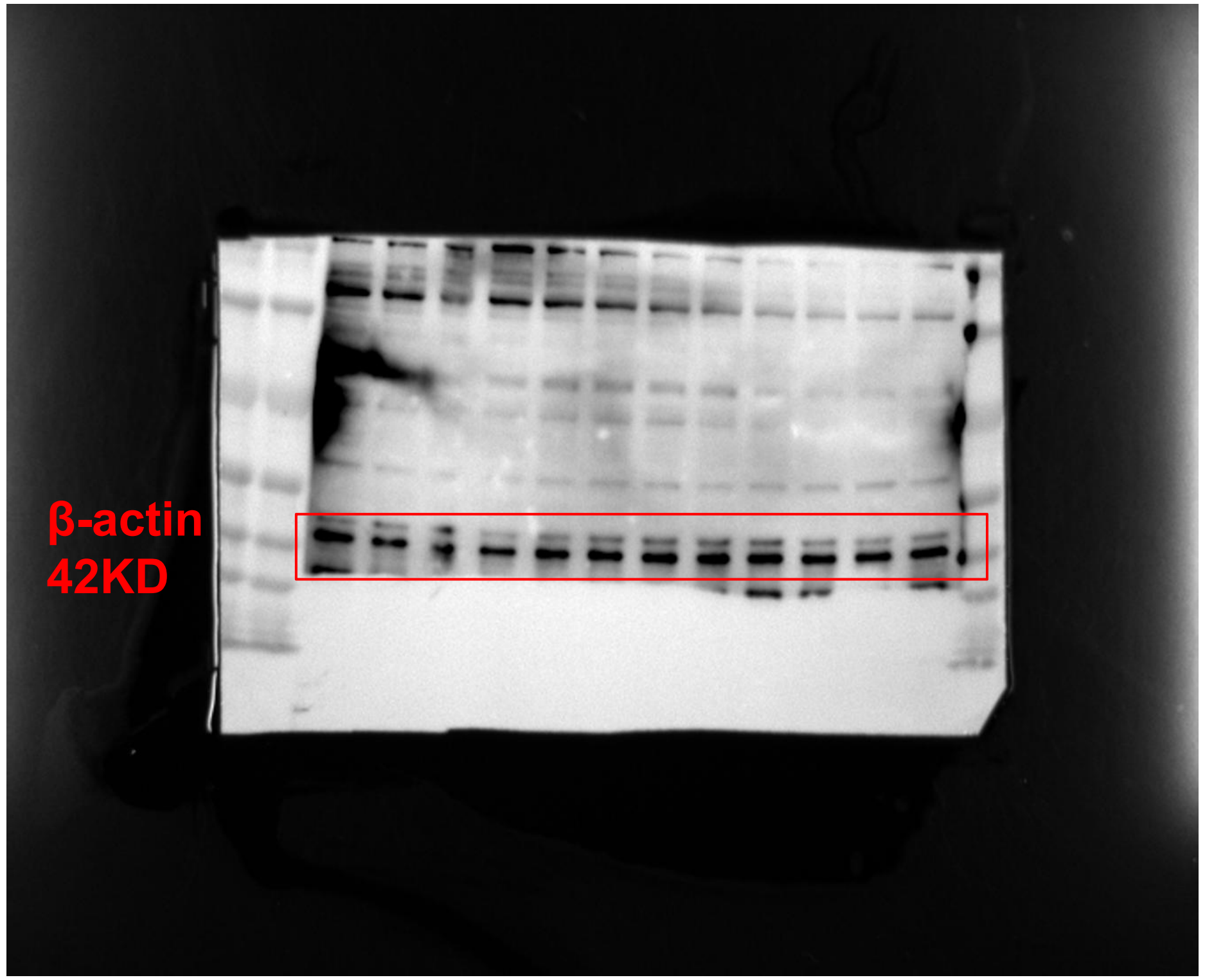


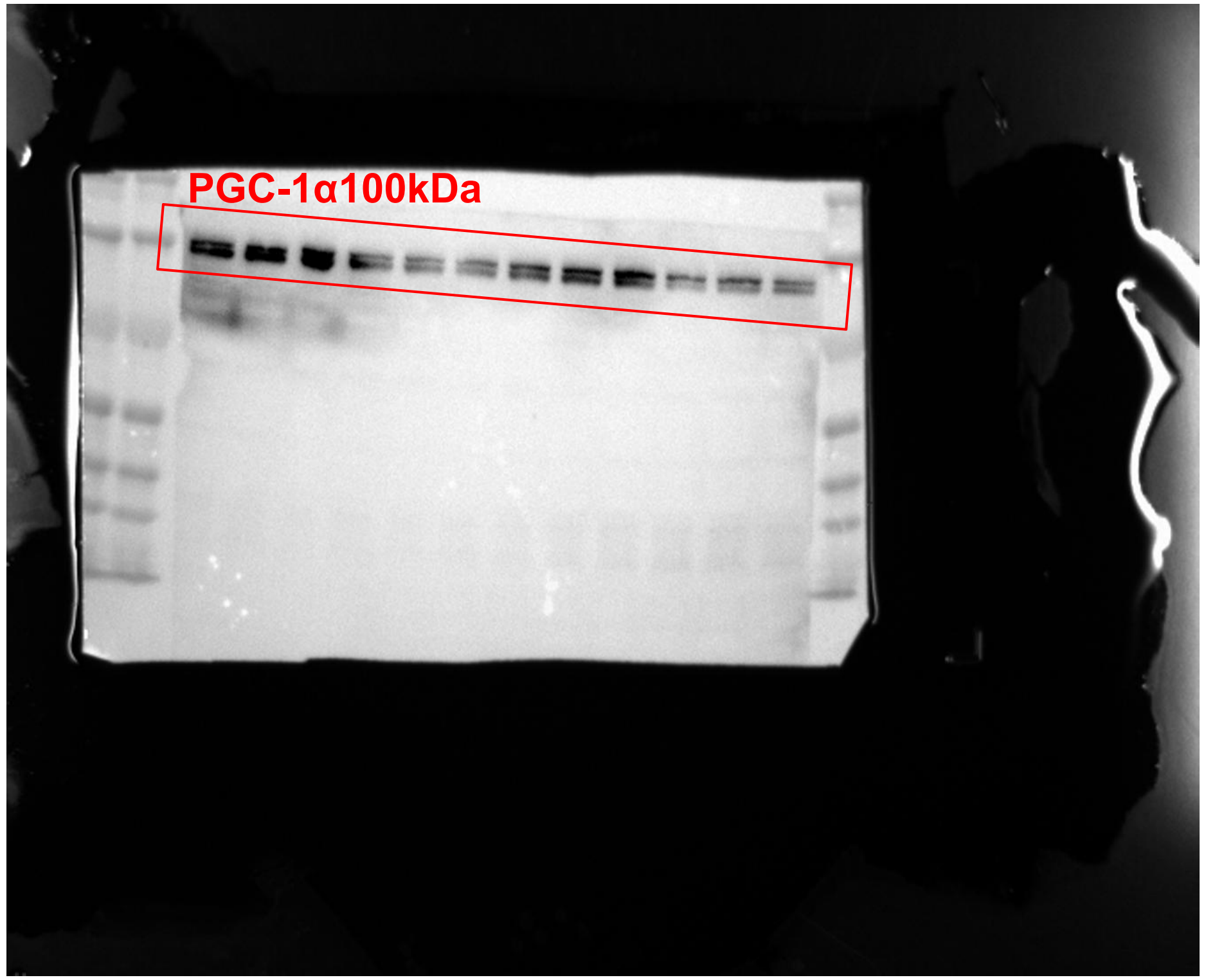

Fig.8E

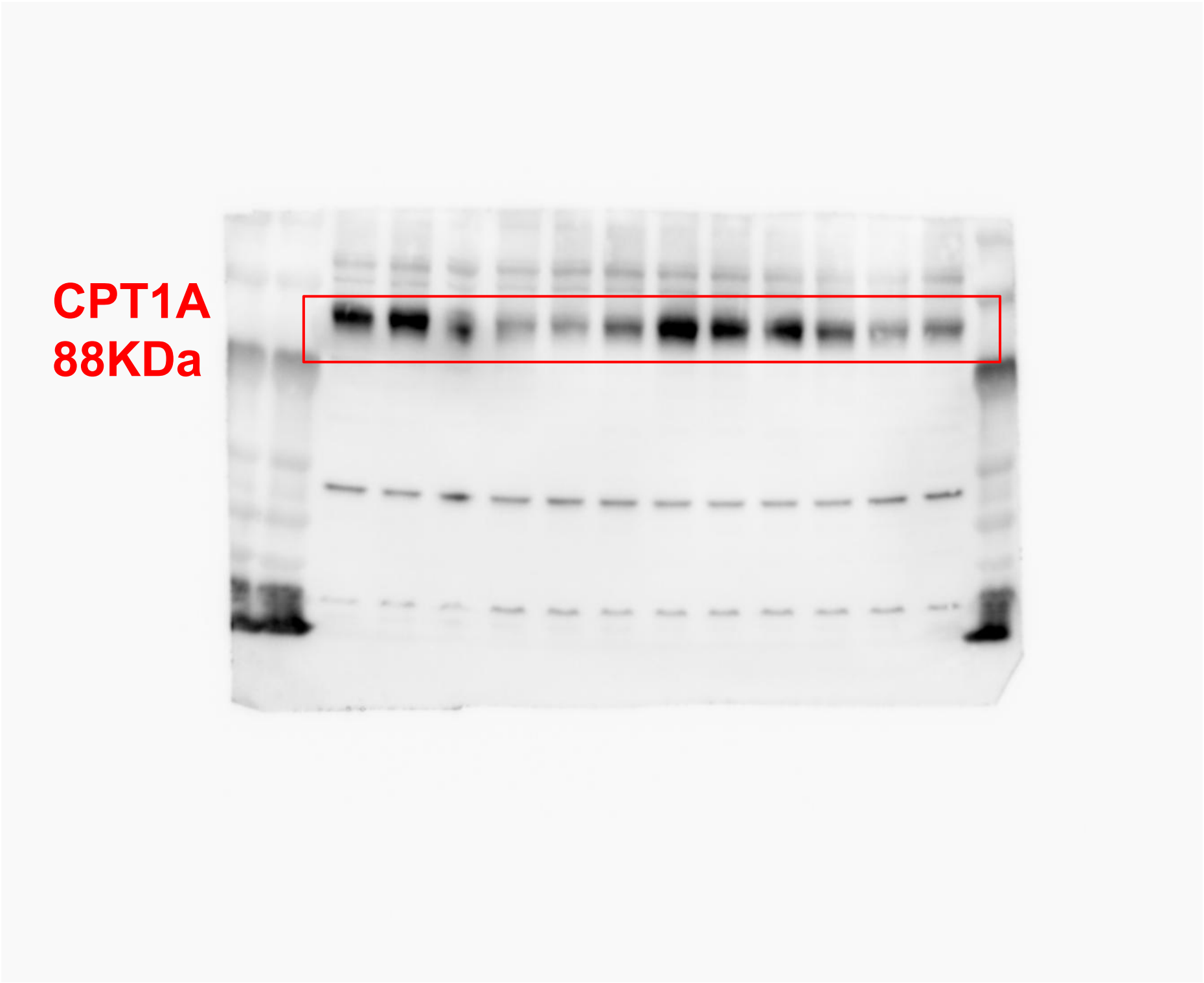


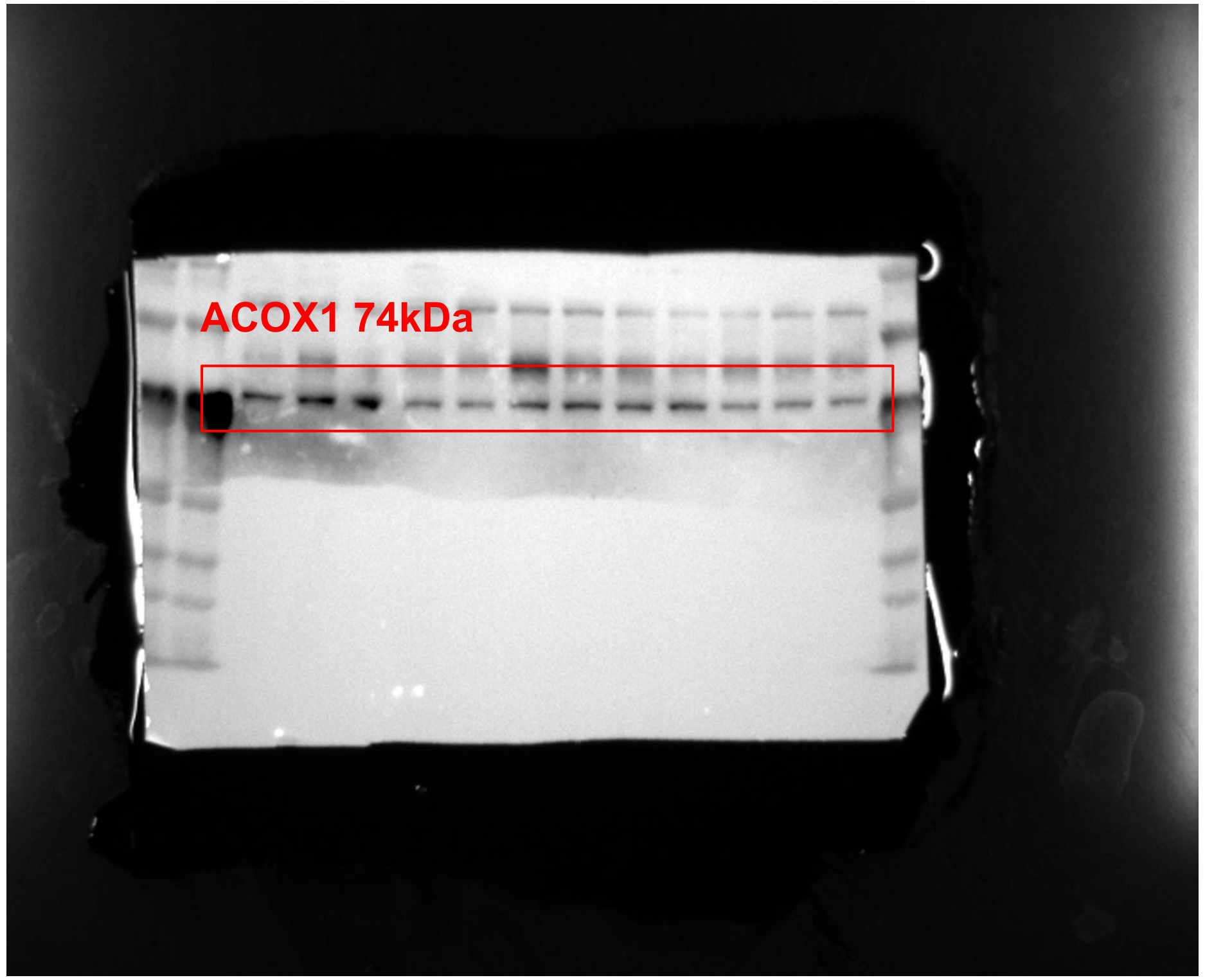

**Fig.8E**

PPARα
53KDa

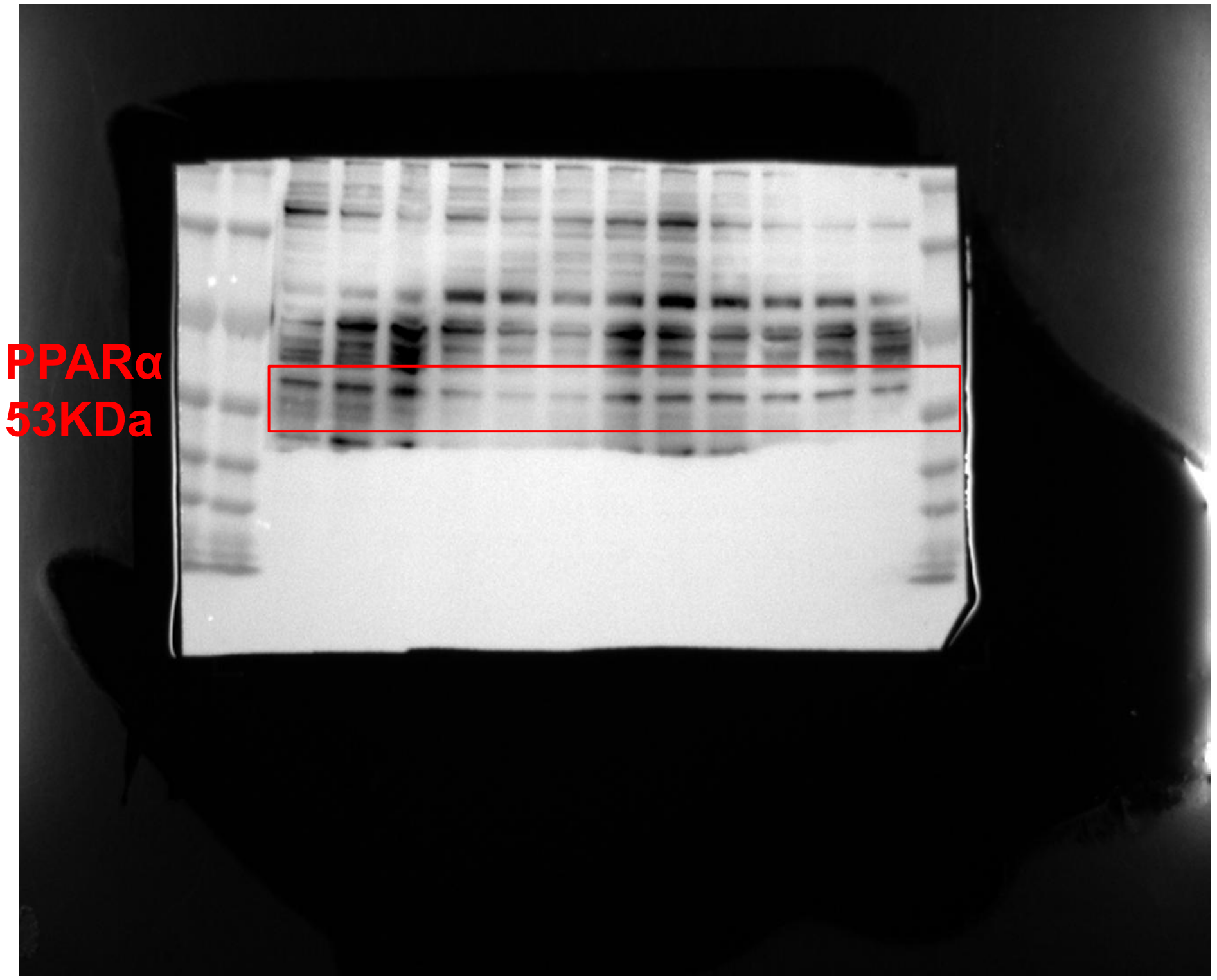

FABP1
15KDa

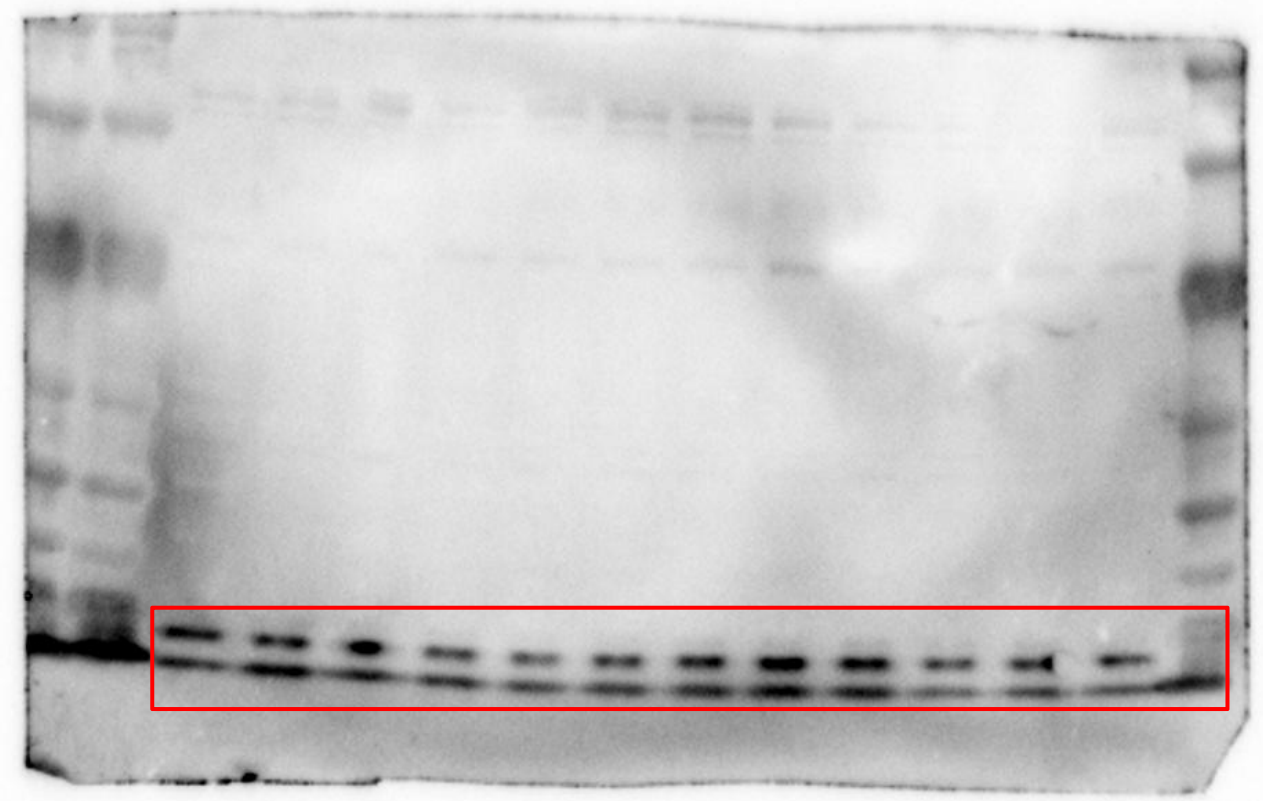

Supplement: Supplementary file 1 [file DataSheet1.pdf]
