# Supplementary material for: Green edible crataegi fructus-derived carbon dots ameliorate hyperlipidemia via the PPARα signaling pathway
Source: Front Pharmacol. 2026 Oct 16;17:1985522. doi: 10.3389/fphar.2026.1985522 (PMC13720364; doi:10.3389/fphar.2026.1985522)
Supplement: Supplementary file 2 [file Supplementaryfile1.docx]

**Supplementary Materials**

1.HPLC

In this study, high-performance liquid chromatography (HPLC) was used to analyze the differences in chemical composition between raw Crataegi Fructus and the CFC-CDs solution. The specific experimental procedure was as follows: After grinding the raw Crataegi Fructus, 10 g of the sample was precisely weighed and placed in a stoppered volumetric flask; 10 mL of methanol was added to bring the volume to the mark; the sample was then subjected to ultrasonic-assisted extraction for 30 minutes (ultrasonic frequency: 40 kHz), yielding a 1 g/mL methanol extract of raw Crataegi Fructus. A 1 g/mL aqueous solution of CFC-CDs, prepared at B °C for Ⅰ h, was also used as a test solution. Prior to detection, the samples were filtered through a 0.22 μm microporous filter membrane to remove impurities, and care was taken to remove air bubbles from the liquid vials containing the samples.

Based on preliminary experiments, the analytical conditions were selected, and the optimized chromatographic system parameters are as follows: A Kromasil C18 reverse-phase column (ZORBAX SB-C18 column, 250 mm × 4.6 mm, 5 μm) was used for separation, and the detector was set to collect signals at a wavelength of 325 nm. The column oven was maintained at a constant temperature of 30 ± 1 °C; the mobile phase was run at a constant flow rate of 1.0 mL·min⁻¹, and the injection volume was controlled at 20 μL. The mobile phase system consisted of acetonitrile (Phase A) and a 0.1% aqueous acetic acid solution (Phase B). The gradient program was set as follows: in the initial stage (0–15 min), the Phase A proportion increased linearly from 5% to 10%; during the middle phase (15–30 min), the gradient increases to 24% Phase A; and during the final phase (30–50 min), the Phase A concentration continues to increase to 40%, achieving efficient separation of the target compounds.

2. Preparation Procedure

Accurately weigh a specific amount of Crataegi Fructus pieces, place them in a crucible, seal it with aluminum foil, cover it, and place it in a muffle furnace. After closing the furnace door, set the muffle furnace’s automatic heating program as follows: In the first stage, rapidly raise the furnace temperature to 70 °C within 5 minutes and maintain it for 25 minutes; Stage 2: Raise the temperature to B °C within 25 minutes and maintain it for Ⅰ hours. After the calcination program ends, wait until the dial display indicates the furnace temperature has dropped to 70 °C, then open the furnace door and allow it to cool to room temperature. Weigh the charcoal powder and calculate the carbonization yield to be 37.5%. After weighing the charred Crataegi Fructus, grind it in a grinder, then add deionized water (DW) equal to 30 times the volume of the charcoal powder and decoct it in a water bath maintained at 100 °C for 1 hour. Repeat the decoction process three times. After each decoction, allow the solution to cool to room temperature, then perform coarse filtration using filter paper followed by fine filtration using a 0.22 μm microporous filter membrane. Collect the filtrates from the three decoctions and concentrate them using a rotary evaporator, Finally, the solution was concentrated to 1 g/mL (mass of Crataegi Fructus charcoal powder per final solution volume), and the concentrated solution was transferred to a ready-to-use dialysis bag with a molecular weight cut-off of 1000 Da. The dialysis bag was placed in a beaker containing DW, and the beaker was then placed on a magnetic stirrer for dialysis. The deionized water in the beaker was replaced at regular intervals each day, and dialysis was continued for at least 7 days until the deionized water outside the dialysis bag became colorless and transparent. The liquid inside the dialysis bag was filtered again using a microporous filter membrane, and the filtrate was collected and stored in a 4 °C refrigerator for later use.

3. Calculation of CFC-CDs Dosage

According to the 2020 edition of the Pharmacopoeia of the People’s Republic of China, the recommended clinical dosage of Crataegi Fructus is 9–12 g/day. Chinese Materia Medica recommends a decoction dosage of 10–15 g, with a maximum of 30 g. Actual dosage should be adjusted based on the patient’s condition. In this study, a dose of 20 g/day was selected (based on a human body weight of 60 kg); converting this to an equivalent dose for rats based on body surface area, the rat dosage was 2 g/kg. An equal dose of raw Crataegi Fructus was carbonized to produce Crataegi Fructus charcoal, with a carbonization yield of 37.5%. Based on the mass of the Crataegi Fructus charcoal, a 1 g/mL CFC-CDs solution was prepared and then freeze-dried at low temperature to obtain CFC-CDs freeze-dried powder. According to calculations, after equivalent conversion from the crude drug to Crataegi Fructus charcoal, the corresponding high dose of CFC-CDs freeze-dried powder administered to rats was 1.88 mg/kg/day. After serial dilutions, the medium and low doses were determined to be 0.94 mg/kg and 0.47 mg/kg, respectively.
